# Study of FoxA Pioneer Factor at Silent Genes Reveals Rfx-Repressed Enhancer at *Cdx2* and a Potential Indicator of Esophageal Adenocarcinoma Development

**DOI:** 10.1371/journal.pgen.1002277

**Published:** 2011-09-15

**Authors:** Jason A. Watts, Chaolin Zhang, Andres J. Klein-Szanto, Jay D. Kormish, Jian Fu, Michael Q. Zhang, Kenneth S. Zaret

**Affiliations:** 1 Department of Cell and Developmental Biology, University of Pennsylvania School of Medicine, Philadelphia, Pennsylvania, United States of America; 2 Cancer Biology Program and Department of Pathology, Fox Chase Cancer Center, Philadelphia, Pennsylvania, United States of America; 3 Cold Spring Harbor Laboratory, Cold Spring Harbor, New York, United States of America; 4 Department of Molecular and Cell Biology, Center for Systems Biology, The University of Texas at Dallas, Richardson, Texas, United States of America; 5 Ministry of Education Key Laboratory of Bioinformatics and Bioinformatics Division, Tsinghua National Laboratory for Information Science and Technology, Tsinghua University, Beijing, China; Fred Hutchinson Cancer Research Center, United States of America

## Abstract

Understanding how silent genes can be competent for activation provides insight into development as well as cellular reprogramming and pathogenesis. We performed genomic location analysis of the pioneer transcription factor FoxA in the adult mouse liver and found that about one-third of the FoxA bound sites are near silent genes, including genes without detectable RNA polymerase II. Virtually all of the FoxA-bound silent sites are within conserved sequences, suggesting possible function. Such sites are enriched in motifs for transcriptional repressors, including for Rfx1 and type II nuclear hormone receptors. We found one such target site at a cryptic “shadow” enhancer 7 kilobases (kb) downstream of the *Cdx2* gene, where Rfx1 restricts transcriptional activation by FoxA. The *Cdx2* shadow enhancer exhibits a subset of regulatory properties of the upstream *Cdx2* promoter region. While *Cdx2* is ectopically induced in the early metaplastic condition of Barrett's esophagus, its expression is not necessarily present in progressive Barrett's with dysplasia or adenocarcinoma. By contrast, we find that *Rfx1* expression in the esophageal epithelium becomes gradually extinguished during progression to cancer, i.e, expression of *Rfx1* decreased markedly in dysplasia and adenocarcinoma. We propose that this decreased expression of *Rfx1* could be an indicator of progression from Barrett's esophagus to adenocarcinoma and that similar analyses of other transcription factors bound to silent genes can reveal unanticipated regulatory insights into oncogenic progression and cellular reprogramming.

## Author Summary

FoxA transcriptional regulatory proteins are “pioneer factors” that engage silent genes, helping to endow the competence for activation. About a third of the DNA sites we found to be occupied by FoxA in the adult liver are at genes that are silent. Analysis of transcription factor binding motifs near the FoxA sites at silent genes revealed a co-occurrence of motifs for the transcriptional repressors Rfx1 and type II nuclear hormone receptors (NHR-II). Further analysis of one such region downstream of the *Cdx2* gene shows that it is a cryptic enhancer, in that it functions poorly unless Rfx1 or NHR-II binding is prevented, in which case FoxA1 promotes enhancer activity. *Cdx2* encodes a transcription factor that promotes intestinal differentiation; ectopic expression of *Cdx2* in the esophagus can help promote metaplasia and cancer. By screening numerous staged samples of human tissues, we show that Rfx1 expression is extinguished during the progression to esophageal adenocarcinoma and thus may serve as a marker of cancer progression. These studies exemplify how the analysis of pioneer factors bound to silent genes can reveal a basis for the competence of cells to deregulate gene expression and undergo transitions to cancer.

## Introduction

The development of a multicellular organism requires the formation of functionally distinct cell types through the differential activation of gene expression. Such gene expression programs are enabled by transcription factors that endow the progenitors with the competence to differentiate under the influence of inductive signals [1]–[6]. During pathogenesis, effectors that damage cells can lead to aberrant induction of gene expression, but in these cases less is known about the transcription factor networks that govern the competence for such changes. In this paper, we describe a means to reveal transcription factor networks that underlie the ability of endoderm-derived tissues to undergo metaplasia, or cell type conversion, during pathogenesis [7].

FoxA transcription factors help establish developmental competence for the endoderm-derived tissues [6], [8]. In the mouse there are three non-allelic FoxA genes, each containing a highly conserved winged-helix forkhead DNA-binding domain [9]. FoxA2 is the earliest to be expressed and is required for endoderm development [10]–[12], while FoxA1 and FoxA2 are redundantly required for liver development [13]. We previously found that a FoxA target site is engaged at the *alb1* gene enhancer in undifferentiated mouse endoderm cells where *alb1* is transcriptionally silent, and occupancy correlates with the potential of the cells to be activated in response to developmental signals [14]–[16]. FoxA factors were further shown to occupy their sites on nucleosomes in compacted chromatin *in vitro* and open a local domain for other factors to bind [17]. This led to the concept of FoxA as a pioneer factor, endowing competence for silent genes to be activated [6]. The pioneer model extends to acute hormone regulation in adult cells, where prior FoxA engagement in chromatin enables estrogen receptor and androgen receptor binding to chromatin and subsequent gene activation [18]–[20].

Another context for silent gene activation is typified by metaplastic transitions, where a cell changes from one type to another. These transitions often occur in response to chronic cellular injury, resulting in pathology. Cdx2 is a homeodomain transcription factor expressed in the developing gut, where it mediates the differentiation of intestinal epithelial cells [21]–[23]. In the adult, *Cdx2* expression is normally restricted to the mid- and hindgut regions; loss of *Cdx2* in the gut leads to expression of an esophageal program [23]. Indeed, chronic esophageal damage from reflux of gastro-duodenal contents can result in the aberrant expression of *Cdx2* in the esophageal foregut epithelium and, together with other molecular changes, promotes metaplasia of the cells to an intestinal phenotype (Barrett's Esophagus) and subsequent adenocarcinoma [24]–[26]. A fundamental question in such circumstances is whether pioneer or competence factors might engage silent genes in normal cells, such as at *Cdx2* in endodermal cells of non-intestinal lineages, thereby potentiating the genes' aberrant activation. If such is the case, there may be regulatory mechanisms that normally restrain such activation. Understanding such networks would provide insight into the basis for diverse pathologies.

To better understand how regulatory factors might endow transcriptional competence for silent genes, as well as how other factors might restrain such competence, we analyzed FoxA2 occupancy in the adult mouse liver, where FoxA factors can occupy silent genes [27], [28]. Using our genomic data, we analyzed transcription factor binding motifs adjacent to FoxA2-bound sites at genes that are silent, separately from motifs at FoxA2-bound sites at genes that are active. We identified binding motifs for the repressors Rfx1 and type II nuclear hormone receptors at the silent FoxA2 gene targets and confirmed protein binding and subsequent repression of adjacent FoxA2 sites at a novel and otherwise silent, *Cdx2* enhancer element in non-intestinal cells. We further investigated the status of Rfx1 in human esophageal epithelium and in different precursor lesions leading to adenocarcinoma. We conclude that FoxA can occupy silent genes whose activation is restricted by locally binding repressors and suggest that perturbation of such networks can help explain cellular changes leading to invasive cancer. Our approach revealed that Rfx1 expression decreases gradually during cellular progression to esophageal adenocarcinoma, indicating that mechanistic and clinically useful insights can emerge from studies of transcription factors bound to silent genes.

## Results

### FoxA2 genomic location analysis

To identify FoxA2 occupied sites at active and inactive genes relevant to endoderm-derived tissue in the adult liver, we designed a high-density tiling microarray covering 210 genes related to endoderm, liver, pancreas, lung, gut, signaling, and cancer (Table S1 and Figure S1A). Each tiled locus included the coding region and its flanking 30 kb upstream and 10 kb downstream sequence, tiled at a density of one 50-nucleotide probe every 24 bp. For regions containing multiple genes of interest, we included larger intergenic regions, such that the final coverage of each of the selected loci ranged from 45–350 kb (Figure S1B). In aggregate, the “endoderm array” covered 14 Mb or 0.5% of the mouse genome. The arrays were masked for repeat sequences.

Next, we isolated chromatin from adult mouse livers which were perfused with formaldehyde *in situ* [29] and sheared to 50–300 bp (Figure S1C). ChIP-qPCR assays with a FoxA2 antibody, comparing liver (FoxA2+) and kidney (FoxA2-) confirmed the antibody specificity (Figure S1D). We pooled the DNA recovered from triplicate immunoprecipitations, amplified the material by ligation-mediated PCR (Figure S1E), and then labeled the DNA and hybridized it to the endoderm array. We performed three competitive hybridizations: FoxA2 ChIP vs. IgG ChIP, FoxA2 ChIP v.s input DNA, and IgG ChIP vs. input DNA. The resulting hybridization signals were analyzed to locate significant sites of FoxA occupancy using a sliding-window based approach similar to MAT [30] and yielded a set of 193 FoxA2 bound sites on the array (p<0.0001; Figure S2A, S2B, red arrows, and Table S2). We tested 33 sites spanning the range of significant scores (putative FoxA2 positive sites) and 35 sites with insignificant scores (putative FoxA2 negative sites) by locus-specific ChIP-qPCR from independent mouse livers. We observed a high concordance between positive regions from ChIP-chip and FoxA2 occupancy from locus-specific ChIP (Figure S2D, S2E) demonstrating the quality of our bound FoxA2 site assignments (specificity = 90%, sensitivity = 81%). Further *de novo* motif analysis of FoxA2 occupied sites revealed an extended *in vivo* derived FoxA consensus sequence that closely matches the TRANSFAC FoxA motif (Figure S2F). In addition, the sequences near the ChIP-chip defined FoxA2 sites showed a much higher degree of local sequence conservation among vertebrates, compared to distal flanking sequences and random loci without ChIP signals (Figure S2G), suggesting that the ChIP-chip defined regions represent elements under strong purifying selection, with possible function. Finally, we compared our peak assignments to those seen in two subsequent whole genome studies [28], [31]. Of their peaks that were covered by our endoderm array, 86% and 90% overlapped with our hybridization probes that gave us at least a 2-fold enrichment of FoxA2 signal over input. Taken together, we generated a high quality set of FoxA2 target sites in adult liver for further analysis.

### FoxA occupancy at silent targets

**Figure 1:**
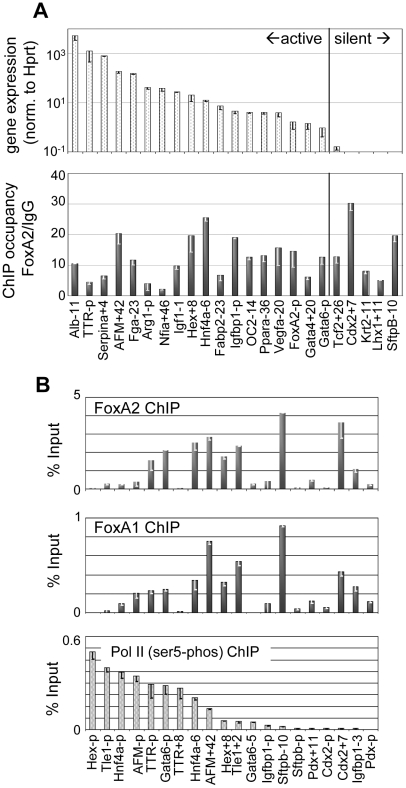
FoxA occupies silent targets in the adult liver. (A) Comparison of liver gene expression as determined by Taqman Low-Density Array to FoxA2 occupancy by ChIP after ranking genes by expression level. For genes with multiple FoxA target sites, the site with the strongest occupancy is indicated. (B) Site-specific ChIP of FoxA2, FoxA1 and Pol II Phospho-serine 5 at ChIP-chip identified targets and nearby control regions in liver. Sites are ranked by degree of Pol II occupancy, with signal as percentage of input after background subtraction.

We quantitatively assessed the relationship between FoxA occupancy and gene activity using Taqman-low-density arrays on 86 of our ChIP-chip positive FoxA target genes, spanning the range of FoxA binding, with liver RNA. Using this information, we partitioned genes that were below one ten-thousandth of *alb1* (*albumin*) expression as weak or silent. By this classification system, 56 genes were highly expressed and 30 genes were weak or silent, demonstrating that FoxA2 clearly occupies transcriptionally silent loci (Figure S3A). Notably, we observed little correlation between the level of gene activity and extent of FoxA occupancy, as measured by ChIP-qPCR of individual genes (Figure 1A). However, FoxA2 binding within 5 kb of the transcriptional start is more frequently associated with active genes under our classification system, whereas more distal elements were equally associated with active or inactive genes (Figure S2B). Taken together, FoxA binds to silent genes in the adult liver, often through distal regulatory elements, as has been observed in other cell types [19].

As an independent metric of gene activity, we performed ChIP against the initiating form of Pol II (ser5-P) and assessed factor occupancy at a set of FoxA target genes covering a broad range of gene expression levels. At each gene we queried for Pol II occupancy at the FoxA target site and at the gene promoter. These sites were ranked by occupancy of active Pol II and tested for occupancy of both FoxA1 and FoxA2 (Figure 1B). We found that there was little correlation between the distribution of Pol II occupancy and the distribution of FoxA occupancy (correlation  = 0.1); whereas there was good concordance between gene activity measured by Pol II occupancy and RNA expression (correlation  = 0.8). Indeed FoxA was strongly bound to genes that are silent by mRNA expression (Figure 1A) and the absence of pol II (Figure 1B), such as at the *Cdx2* gene. We also found that FoxA1 and FoxA2 have a remarkably similar occupancy distribution (Figure 1B), suggesting that the factors share a large set of *in vivo* binding sites. We conclude that FoxA1 and FoxA2 can redundantly occupy a large set of target sites at both active and silent genes in the liver.

### Transcription factors co-associated with FoxA at silent genes

**Figure 2:**
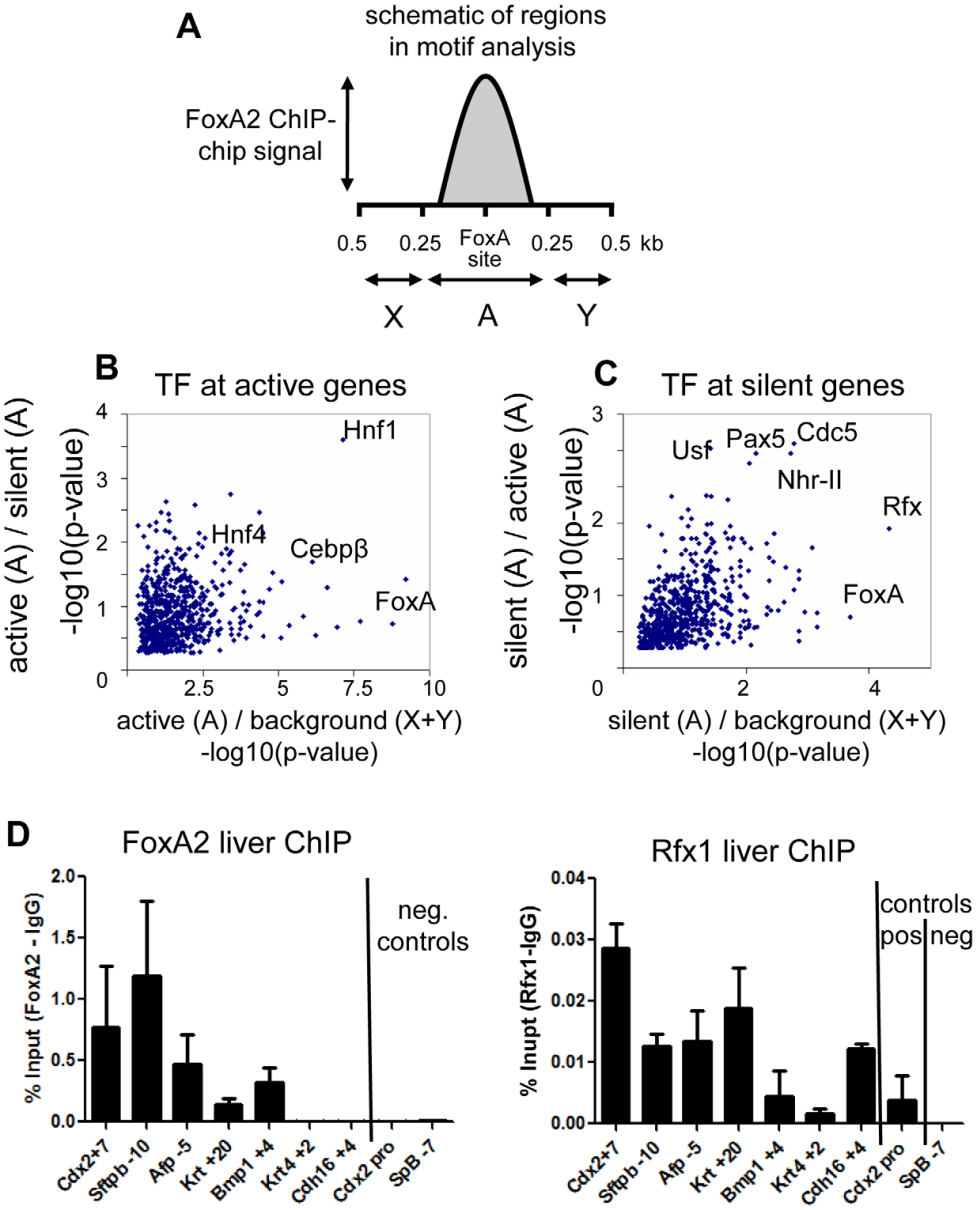
FoxA co-interacting factors. (A) Schematic representation of the ChIP-chip signal at a FoxA bound site, with the FoxA element taken as position 0. Region “A” is the 250 bases flanking the FoxA site and is considered the foreground in motif analysis. Regions “X” and “Y” are the flanking sequence 250-500 bases form the FoxA site and constitute the background. (B) Two-dimensional motif significance of active genes. Each point is a motif with –log10(p-value) shown. X-axis is the p-value from foreground (region “A”) vs background (region X+Y) comparison. The Y-axis is the p-value from comparison of motifs in region A for active genes compared to region A for silent genes. (C) Two-dimensional motif significance of silent genes. The X-axis is as described for panel B. The Y-axis is the p-value from comparison of motifs in region A for silent genes compared to region A for active genes. (D). ChIP against FoxA2 and Rfx1 at selected sites expressed as signal after subtraction of IgG control (n = 3). SpB -7 serves as a negative control site.

To understand how factors co-bound at silent genes could modulate FoxA activity, we performed motif analysis to identify transcription factors which segregate with FoxA at active versus silent genes. This was facilitated by the resolution our tiling analysis, which allowed the direct identification of FoxA binding sites (Figure S2F). We screened 673 TRANSFAC/JASPAR vertebrate motifs of known transcription factors at FoxA targets in two dimensions (see Materials and Methods). In the first dimension, we identified motifs enriched near FoxA bound sites compared to adjacent unoccupied sequences (Figure 2A–2C). In the second dimension, we identified motifs enriched near active FoxA sites compared to silent FoxA sites (Figure 2B), or vice versa (Figure 2C). At active genes there was an enrichment of transcription factor motifs known to be important for liver-specific gene expression, including HNF4a, C/EBP, and HNF1 (Figure 2B) [32], [33]. In contrast, the transcription factor motifs enriched at silent genes included those for Rfx (motif M00975), type II nuclear hormone receptors (motif M00964), USF (motif M00217), PAX5 (motif M00143), and CDC5 (motif M00478) (Figure 2C). The Rfx factors are a family of transcriptional repressors conserved from yeast to mammals, with seven members in mammals [34], [35]. Rfx1 is expressed in most cells, including in the liver, and has been identified as the Rfx family member most functionally related to the yeast repressor Crt1 [35]. We tested a subset of sites identified by bioinformatics analysis and used ChIP-qPCR to confirm co-occupancy of FoxA2 and Rfx1 at 5 of 7 sites tested (Figure 2D), compared to a negative site control. This suggested a model where the intrinsically positive action of FoxA at silent genes could be counterbalanced by a repressive action of Rfx1.

### Novel enhancer at the Cdx2 locus

**Figure 3:**
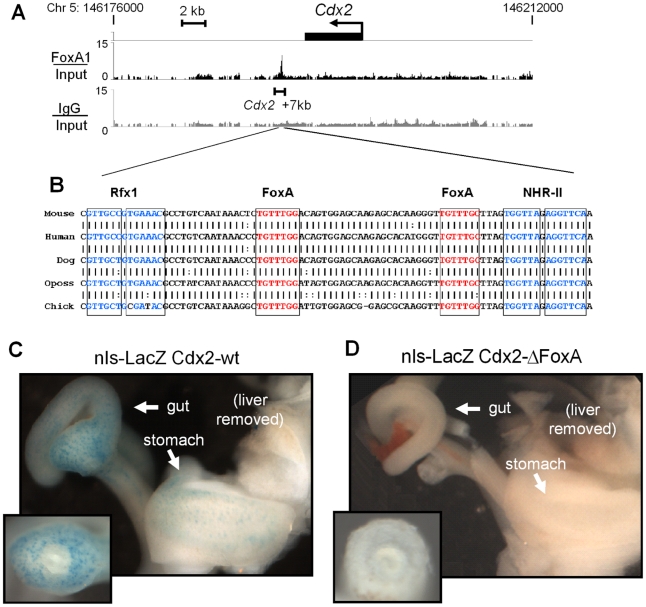
Novel FoxA binding site at *Cdx2*. FoxA2 ChIP-chip signal at the *Cdx2* locus on mouse chromosome 5. Signal from FoxA2 vs. input (black) and IgG vs. input (grey) are shown on a linear scale. Below is an expanded view of the DNA sequence of the region within ChIP-chip peak, depicting sequence conservation between mouse and chicken. Transcription factor binding sites for FoxA, Rfx, and NHR-II are indicated. (C and D) LacZ staining of nuclear-β-galactosidase reporter mice at E12.5. Embryos containing reporter driven by the wild-type *Cdx2* enhancer (C) and FoxA-site mutants (D). Cross sections through hindgut (insets).

To investigate the regulatory interactions between FoxA and Rfx at silent genes, we focused on *Cdx2* as a candidate gene. *Cdx2* is transcriptionally silent in the liver both based on its lack of mRNA expression and lack of Pol II occupancy at its promoter (see Figure 1A and 1B). *Cdx2* is ectopically expressed in various pathologic conditions, including Barrett's esophagus and biliary cancer [24]–[26], so understanding its regulation is clinically relevant. Our ChIP-chip analysis revealed a strong peak of FoxA2 at a previously uncharacterized element 7 kb downstream of the *Cdx2* transcription start site (TSS) (Figure 3A; see 'FoxA/Input" track; bracketed region). This new element contains a 156 bp sequence which exhibits greater than 78% sequence conservation from mammals to birds and extends to about 500 bp overall (Figure 3B and data not shown). Within this conserved region there is an 83 bp sequence containing two FoxA sites, an Rfx site, and a direct repeat consensus (DR1) for type II NHRs [36]–[39].

Previous work showed that the *Cdx2* promoter and upstream regulatory sequences are sufficient to drive expression in colon cells [40]–[42], so we sought to similarly assess the *in vivo* function of the FoxA-bound conserved element 7 kb downstream of *Cdx2*. Endogenous *Cdx2* is initially expressed in the caudal region of the embryo both in the distal gut tube and in the unsegmented parasomitic mesoderm, and by day 12.5 of embryonic development, *Cdx2* expression is largely restricted to the developing gut tube [21], [22], [41]. We made stable transgenic mice containing a nuclear β-galactosidase reporter driven by a 500 bp fragment spanning the *Cdx2* +7 kb element (LacZ-wt) or the *Cdx2* +7 kb element with both FoxA sites mutated (LacZ-ΔFoxA). When tested in E12.5 embryos, the LacZ-wt reporter line exhibited punctate nuclear LacZ staining beginning in the colon and extending caudally to the stomach, where scattered cells were positive (Figure 3C). We did not observe LacZ staining in the gut tube anterior to the stomach, nor was staining visible in the liver or the lung, mimicking the endogenous *Cdx2* pattern. However, the transgene did express LacZ in other embryonic tissues, perhaps due to position effects (data not shown). In contrast, two independent LacZ-ΔFoxA lines containing the same transgenic sequences, except for clustered point mutations of the FoxA sites, failed to express LacZ in the gut (Figure 3D) but retained non-specific expression (data not shown). These results demonstrated that the *Cdx2* +7 kb element is a weak tissue-specific enhancer that is dependent upon FoxA binding sites for activity in developing embryos.

### Rfx sites restricting Cdx2 +7 kb enhancer function

**Figure 4:**
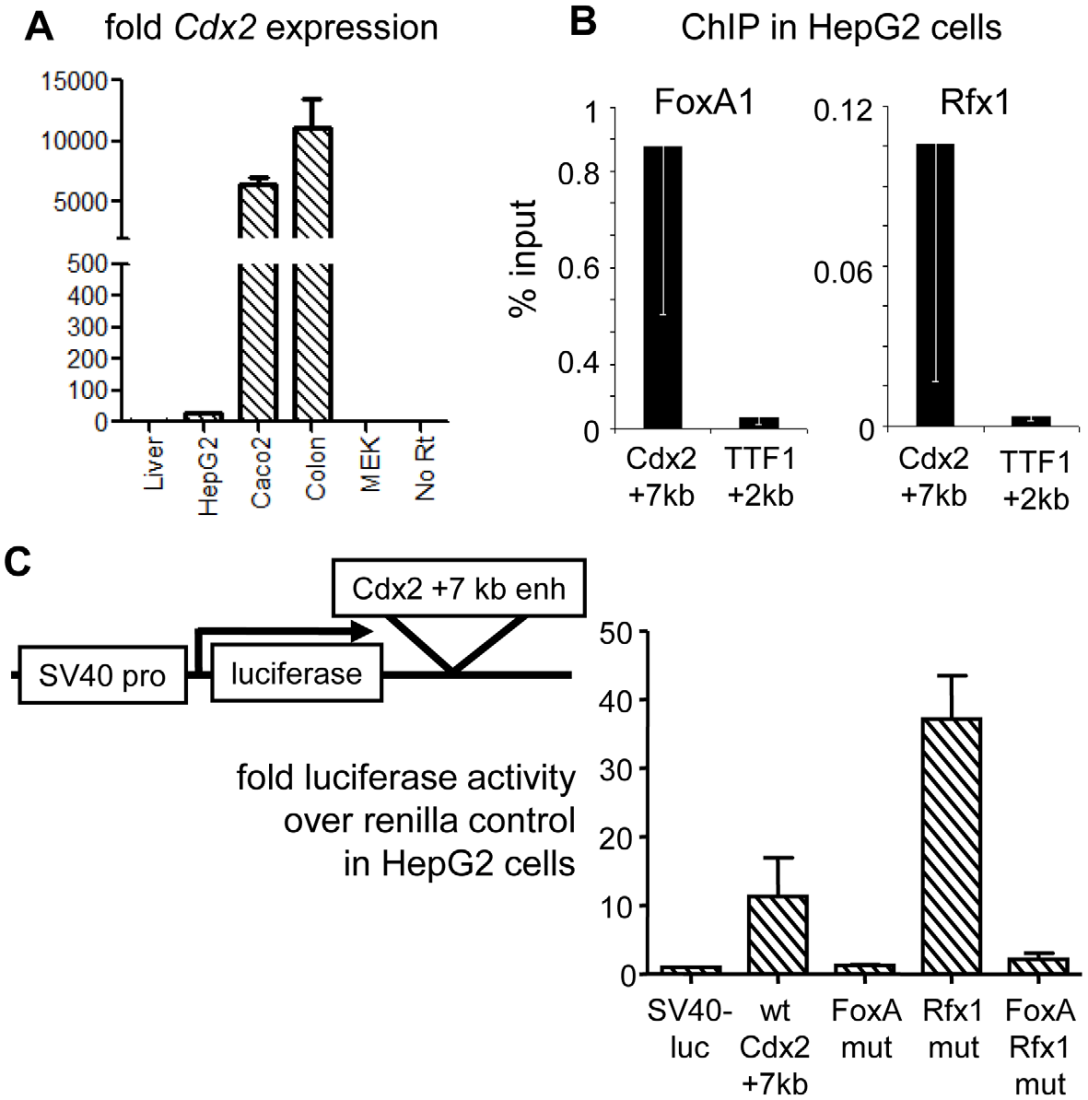
Genetic interactions at the Cdx2 +7 kb enhancer. (A) Endogenous Cdx2 expression in mouse liver, HepG2 cells, Caco-2 cells, mouse colon and MEK cells, indicating the poor expression in HepG2 cells used for transfection. (B) ChIP for FoxA1 and Rfx1 in HepG2 cells comparing occupancy at Cdx2 +7 kb and the negative control site TTF1 +2 kb. (C) Dual-reporter luciferase assays using the Cdx2 element, with combinations of FoxA and Rfx1 site mutations. Cells were co-transfected with pRL-CMV-renilla and the indicated pGL3 reporter plasmids. Luciferase activity expressed as fold over the empty pGL3 vector after normalization to CMV-renilla in arbitrary units.

We next sought to determine the role of the Rfx sites in the regulation of the *Cdx2* +7 kb element. Upon scanning endogenous *Cdx2* expression levels in cell lines, we found that HepG2 liver carcinoma cells [43] express very low *Cdx2* levels compared to the Caco2 colon cancer line or mouse colon; though the gene was expressed about 10-fold above the negative background seen in liver (Figure 4A). Still, ChIP of the conserved +7 kb element (Figure 3B) in HepG2 cells showed strong FoxA and Rfx1 binding, as seen in mouse liver (Figure 2D), compared to a negative control site (Figure 4B).

We then created a series of luciferase reporter constructs with a wild type copy of the 500 bp+7 kb element, as well as variant elements with clustered mutations of the FoxA or Rfx sites, inserted downstream of the reporter (Figure 4C). In three independent HepG2 transfection experiments, each quantified in duplicate, the wild-type *Cdx2* +7 kb enhancer elicited a ten-fold greater activity than the control plasmid in HepG2 cells (Figure 4C, “wt Cdx2 +7 kb”). Mutation of the FoxA sites resulted in loss of enhancer activity (Figure 4B, “FoxA mut”), consistent with our results in transgenic mice. Strikingly, mutation of the Rfx site resulted in an increase in reporter activity, compared to the wild type element, demonstrating that factors that bind the site repress the *Cdx2* enhancer (Figure 4B, “Rfx1 mut”). Simultaneous mutation of both the Rfx and FoxA sites resulted in a loss of enhancer activity, indicating that FoxA binding is necessary for the cryptic activity when the Rfx1 site is lost. Using *Cdx2* as a representative, we suggest that FoxA is able to functionally engage a silent or very poorly expressed gene and that its potential stimulatory effect on gene activity can be attenuated by Rfx1-mediated repression.

### Progressive Rfx1 loss in esophageal adenocarcinoma

**Figure 5:**
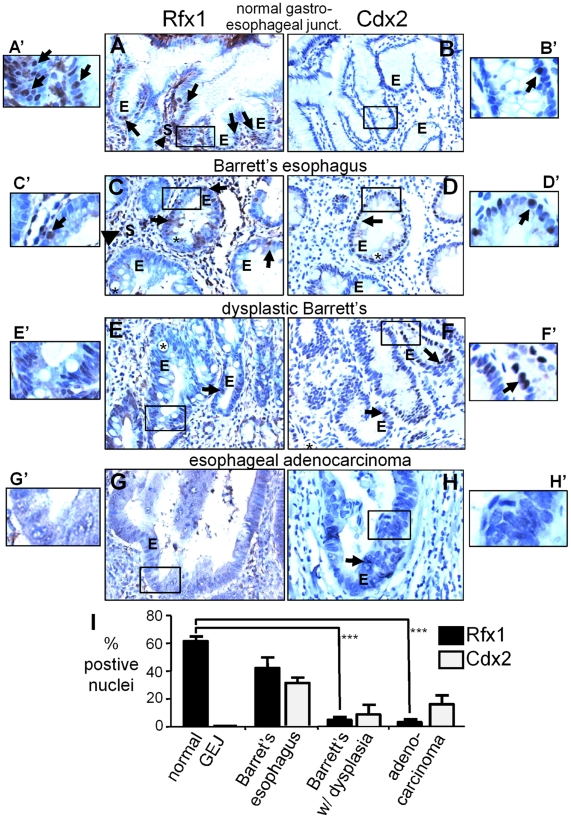
Rfx1 downregulation in esophageal adenocarcinoma. (A–H) Immunohistochemisty of Rfx1 and Cdx2 expression in: (A, B) normal glandular epithelium of the gastro-esophageal junction, (C, D) Barrett's esophagus, (E, F) Barrett's esophagus with dysplasia, and (G, H) esophageal adenocarcinoma. Arrows indicate Rfx1 or Cdx2 positive nuclei in the epithelium (E); arrowheads indicate Rfx1 positive nuclei in the stroma(s). Asterisks indicates large secretory vacuoles characteristic of intestinal metaplasia (Barrett's esophagus). (A′–H′) higher magnification views of the regions boxed in A–H. (I) Graph of the average percentage of Rfx1 positive nuclei at the indicated stages. Error bars indicate the standard error of the mean. The difference in Rfx1 expression between normal and Barrett's esophagus without dysplasia is not statistically significant (p≤0.12); whereas the difference in Rfx1 expression between normal and Barrett's esophagus with dysplasia, and normal and adenocarcinoma are significant (p≤0.0001 by the Student's t-test).

Inappropriate activation of *Cdx2* is one of the initiating events in the progression of normal esophageal mucosa to the development of Barrett's esophagus [24]–[26], where esophageal cells form columnar cells that morphologically resemble those of the intestinal epithelium. Patients with Barrett's esophagus are at increased risk to develop dysplastic Barrett's esophagus and then esophageal adenocarcinoma [44]. Since we found that Rfx1 is a negative regulator of the *Cdx2* +7 element, we wanted to determine if Rfx1 levels change during the progression from normal human esophageal mucosa to the development of esophageal adenocarcinoma. Accordingly, we performed immunohistochemistry on tissue microarrays containing anonymized patient-derived samples from the normal glandular mucosa at the gastro-esophageal junction (n = 5), Barrett's esophagus (n = 11), dysplastic Barrett's (n = 6), and invasive esophageal adenocarcinoma (n = 20) (Figure 5A–5H). The dysplastic Barrett's samples were from patients who had already developed esophageal adenocarcinoma. We confirmed antibody specificity by staining adjacent sections with Rfx1 antibody alone or in the presence of blocking epitope peptide, and observed a loss of positive nuclear staining in the presence of blocking peptide in both the epithelial and stromal cells (Figure S4). In addition, we stained contiguous serial sections for Cdx2.

In the normal glandular epithelium of the gastro-esophageal junction, we found that Rfx1 is expressed in the nuclei of the epithelial cells lining the esophageal lumen (luminal epithelium; not shown) and the deeper secretory portions of the glandular epithelium (Figure 5A, "E", arrows). We also observed Rfx1 positive nuclei in the surrounding stromal cells (Figure 5A, “S”, arrowhead). In nearby sections, Cdx2 expression was either very sparse or undetectable (Figure 5B). We conclude that Rfx1 is expressed in normal gastroesophageal junction-glandular tissue, whereas Cdx2 expression is marginal or non-existent. In Barrett's esophagus, comparing expression from 11 samples (Table 1), there was a qualitative but not statistically significant decrease in the percentage of Rfx1 positive nuclei in comparison to normal samples (p≤0.12) (Figure 5C, 5I; “E”, arrows). Cdx2 was activated in many of the Barrett's samples (Figure 5D, 5I). However, as noted previously [24]–[26], in Barrett's esophagus with dysplasia and in esophageal adenocarcinoma, the percent of Cdx2 positive cells declined in most samples, but was persistent in others (Figure 5F, 5H, 5I).

**Table 1:** Summary of Rfx1 immunohistochemistry results.

	Samples	Total Nuclei		Stained Nuclei		% Positive Nuclei	
	N =	Mean	S.E.M.	Mean	S.E.M.	Mean	S.E.M.
A. Norm. gland. GEJ	5	351.0	57.8	219.8	41.6	61.6	3.3
B. Barrett's Esophag.	11	305.0	50.7	139.1	52.5	42.2+	7.6
C. Dyspl. Barrett's	6	439.7	33.1	20.2	8.8	4.8†	2.0
D. Adenocarcinoma	20	387.1	42.3	11.6	7.0	3.3‡	2.0

% Rfx1 positive nuclei between (A) normal glandular gastro-esophageal junction and: (B) ^+^Barrett's Esophagus p≤0.12, (C) ^†^Dysplastic Barrett's p≤0.0001, or (D) ^‡^adenocarcinoma p≤0.0001. Of the 20 adenocarcinoma samples in (D), two outliers had 16% and 38% of their nuclei expressing Rfx1 and 18 samples expressed Rfx1 in only 0.6%.

By contrast, in samples of Barrett's esophagus with dysplasia, we found a more marked, statistically significant decrease in the percentage of Rfx1 positive nuclei in epithelial cells (p ≤0.0001) (Figure 5E, 5I, arrows; Table 1), whereas the stromal cells retained Rfx1 expression. The percentage of Rfx1 positive nuclei in adenocarcinoma also decreased relative to normal esophageal tissue, with virtually no staining of the epithelial cells in 18 of 20 samples (p≤0.0001) (Figure 5G, 5I; Table 1) and sporadic staining of the stroma. To confirm these data, we performed Western blot analysis on a nuclear lysate from the H520 human cancer cell line (positive control) and on cell lysates from six anonymized, esophageal adenocarcinoma samples. The blots were also probed to GAPDH to confirm protein loading. As seen in Figure S5, only the positive control cell line exhibited Rfx1 expression, suggesting consistent down-regulation of Rfx1 in the tumor samples. Thus, we conclude that Rfx1 expression in the esophageal epithelium is very frequently lost during the progression to esophageal adenocarcinoma, and its loss appears to be a more reliable marker of cancer progression than the up-regulation and then down-regulation of Cdx2 (Figure 5I).

**Figure 6:**
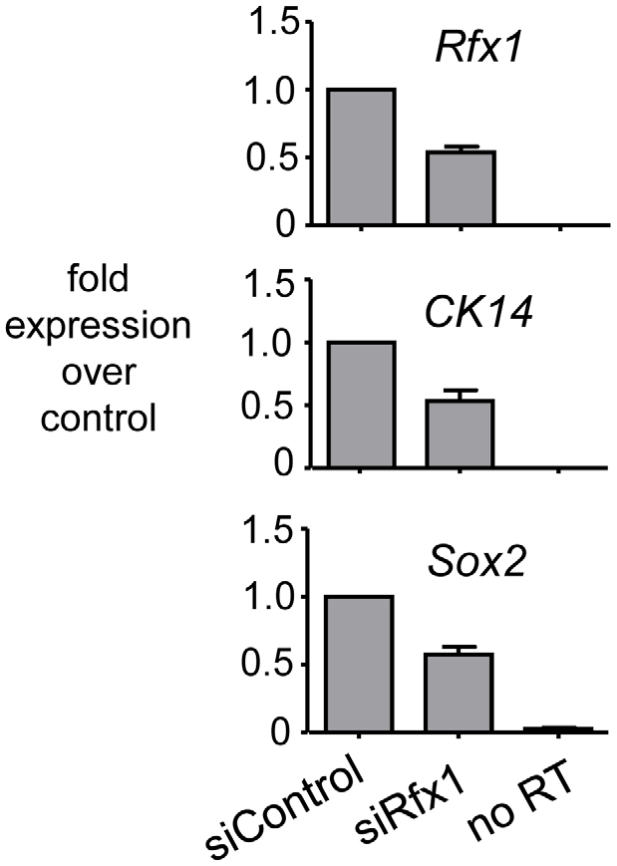
Knock-down of *Rfx1* causes diminution of *CK14* and *Sox2* expression. The panels show qRT-PCR data of RNA from MEK cells treated either with control siRNA, siRNA to *Rfx1*, or the latter but without addition of RT to the reaction. The *Rfx1, CK14,* and *Sox2* genes were investigated. The data indicate that diminution of *Rfx1* causes a similar diminution of the *CK14* and *Sox2* expression in esophageal epithelial cells.

To assess the functional consequences of Rfx1 loss in nontransformed esophageal epithelial cells, we performed siRNA knockdown of Rfx1 in a mouse esophageal keratinocyte (MEK) cell line [45]. The MEK line is derived from the basal cell layer of the squamous epithelium lining the esophagus, proximal to the gastro-esophageal junction [45]. The basal cells of squamous epithelia characteristically express high levels of *Sox2* and cytokeratin *CK14* [46]. siRNA experiments in these and other cells led to, at best, a 50% reduction in *Rfx1* gene expression levels (Figure 6A). The inability to obtain better knock-down seems likely due to *Rfx1* autoregulation (repression) of its own promoter [35]. Thus, as the factor's mRNA is knocked down, its gene's transcription would go up. Under our best *Rfx1* knock-down conditions, we did not observe induction of *Cdx2* expression (data not shown), indicating that the 50% loss of *Rfx1* is insufficient for *Cdx2* activation. Notably, though, upon *Rfx1* knock-down we observed a 50% decrease in the *CK14* and *Sox2* mRNA levels (Figure 6). These findings demonstrate that *Rfx1* helps maintain the expression of genes, presumably indirectly, that contribute to MEK cell differentiation. Taken together with our observation that there is a marked decrease of *Rfx1* expression in the epithelium of Barrett's esophagus with dysplasia, our findings suggest that *Rfx1* helps control the maintenance of the squamous epithelial cell identity in the esophagus and hence antagonizes dysplasia.

### Role of Type II Nuclear Hormone Receptor motif at the +7 kb Cdx2 enhancer

**Figure 7:**
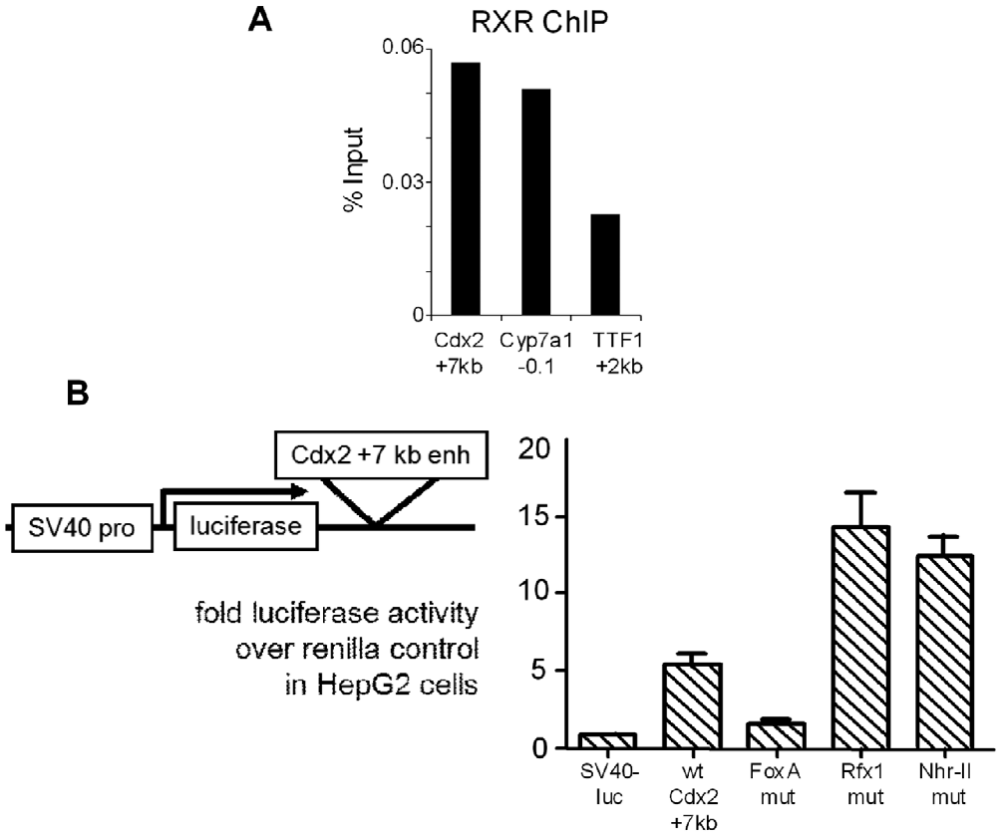
Repressive NHR-II factor binding to the *Cdx2* +7 kb enhancer. (A) ChIP for RXR in HepG2 cells showing occupancy at the *Cdx2* +7 kb enhancer comparable to that at the positive control site at the *Cyp7a1* promoter, versus the signal at the negative control *TTF1* +2 kb enhancer. (B) Dual-reporter luciferase assays using the *Cdx2* element, with combinations of FoxA, Rfx1, and NHR-II site mutations. Cells were co-transfected with pRL-CMV-renilla and the indicated pGL3 reporter plasmids. Luciferase activity expressed as fold over the empty pGL3 vector after normalization to CMV-renilla in arbitrary units. The overall transfection efficiency of this experiment was lower than that seen in Figure 4.

The autoregulation of Rfx1 (35) and inefficiency of siRNA knockdown led us to investigate other parameters affecting the activity of the +7 kb *Cdx2* enhancer. There are a large number of type II nuclear hormone family members which can bind to DR1 motifs, such as those seen at *Cdx2* (Figure 3B). Type II nuclear hormone receptors (NHR-II), formerly orphan receptors, are a class of transcription factors whose activating ligands are metabolic products, such as bile acids [47], and that function as repressors in the absence of ligand [48]. Indeed, bile acids and acidic culture conditions can cause ectopic expression of *Cdx2* [49]. Since type II nuclear hormone receptors form obligate heterodimers with RXR, we performed ChIP against RXR in HepG2 cells. We observed RXR enrichment at the *Cdx2* +7 kb element as compared to a control site in the *TTF1* gene (Figure 7A). The extent of RXR enrichment at the *Cdx2* +7 kb element was similar to that at the *Cyp7a1* promoter, which contains a known type II nuclear hormone receptor binding site [50], [51]. Furthermore, mutation of the NHR-II site in the *Cdx2* +7 kb enhancer resulted in a clear increase in enhancer activity, comparable to that seen with the Rfx1 mutation (Figure 7B). However, we were not able to identify the heterodimeric receptor partner for RXR at the NHR-II element in HepG2 and other cell contexts, and the effects of bile acids on the activity of the *Cdx2* +7 kb element in HepG2 and MEK cells were inconsistent. Regardless, the repressive role of the NHR-II site emphasizes the redundant nature of repression of *Cdx2* and the likely multistep nature required for ectopic gene activation. Furthermore, RXR at the repressive NHR-II domain of the +7 kb enhancer demonstrates how the FoxA pioneer factor bound to silent genes can be restrained by different types of transcriptional repressors.

## Discussion

In contrast to other genomic studies of transcription factor occupancy at active genes, here we focused on the interactions of FoxA with silent target genes. Such an approach allowed us to identify an element near the silent *Cdx2* gene as a cryptic FoxA target. This is notable because *Cdx2* is activated during esophageal metaplastic changes, thereby facilitating, in a percentage of cases, the progression towards adenocarcinoma development [24]–[26], [52]. We employed a two-dimensional motif analysis to identify transcription factors which may function with FoxA at silent genes like *Cdx2*. By this approach, we found that the repressor Rfx1 can restrict the activation of the cryptic FoxA target sequence. We suggest that FoxA occupancy of silent genes in differentiated tissues can endow the competence of such genes to be activated aberrantly and may contribute to human disease. Indeed, FoxA1 has been implicated as a tumor promoting factor [53], [54] and is upregulated in a subset of esophageal cancers as a result of genomic amplification [55]. In our studies, partial knock-down of Rfx1 was insufficient to alter FoxA1 levels (data not shown).

We observed a marked and statistically significant decrease in the levels of Rfx1 expression in Barrett's esophagus with dysplasia and in esophageal adenocarcinoma. Since most Barrett's esophagus lesions do not become dysplastic, it is useful to possess markers that are indicative of the dysplastic transition. We therefore suggest that Rfx1 loss will function as such a marker, and more reliably so than the induction of Cdx2 in earlier stage Barrett's and then its variable down-regulation during progression [24]–[26]. Indeed, recent experiments suggest that Cdx2 plays a relatively early role during progression [56], [57], and thus its expression pattern may be less informative for the later stages. With regard to the small fraction of adenocarcinomas that retain Rfx1 expression, we suggest that either Rfx1 loss is not absolutely necessary for such progression or, like many other genes that are involved with cancer, the samples could, for example, contain point mutations that are not reflected in changes in protein abundance. Many well-known cancer markers appear in a frequency comparable to or less than those in our initial studies of Rfx1. For example, a meta-analysis showed that K-ras mutations progress from an occurrence in 36–44% of early stage pancreatic neoplasias to 87% in later stage cancers [58]. Mutations in p53 occur in frequencies of 25–80% in various cancers tested [59] and mutation in APC occur in up to 83% of colorectal tumors tested [60]; in both cases using tissue sample numbers comparable to those used here. Given the difficulties in qualitative assessment of tissue morphology, specific molecular markers such as Rfx1 can be of high utility in the diagnosis of cancer and its precursors.

Further studies are required to assess whether Rfx1 has a direct tumor suppressive role in the esophageal epithelium. Rfx1 functions genetically downstream of ATR and contributes to the DNA damage response and stalled DNA replication [35]. In response to DNA damage, Rfx1 binding to DNA is lost, leading to the activation of many of its target genes, such as its own promoter and the ribonucleotide reductase gene [35]. Rfx1 also binds the genes for PCNA and c-Myc, thus loss of Rfx1 binding or expression could promote increased cell proliferation [34], [61], [62]. Rfx1 expression was found to be down-regulated in gliomas as a result of promoter methylation and the reintroduction of Rfx1 in transfected glioma cells resulted in decreased cell proliferation, suggesting that Rfx1 may play a role as a tumor suppressor in glioma tumorigenesis [63]. Given these extensive activities of Rfx1 in cell growth and oncogenesis, our discovery of the gradual loss of Rfx1 expression in the progression to esophageal adenocarcinoma suggests that the factor has a functional role in that context. Considering the poor clinical prognosis of esophageal adenocarcinoma [64], Rfx1 down-regulation during progression to adenocarcinoma may be a useful new marker of cancer development. Mechanistically, Rfx1 is a winged helix factor [65], [66] and therefore, like FoxA, could be normally bound to silent chromatin by virtue of intrinsic chromatin binding properties.

By taking a genomic view of FoxA occupancy and focusing our analysis on the interactions at silent target genes, we have uncovered novel gene regulatory interactions at a cryptic enhancer. We found that the +7 kb *Cdx2* enhancer weakly recapitulates part of the developmental activity of the upstream promoter region (Figure 3C), but only the +7 kb element binds FoxA in liver cells, not the *Cdx2* promoter (Figure 3A). We suggest that the +7 kb element functions as a shadow enhancer, similar to those recently discovered in Drosophila [67], [68]. As an additional regulatory element at a gene, possibly arising by sequence duplication, a shadow enhancer may ensure a more precise gene expression pattern in development or allow a new regulatory function to evolve, while other regulatory elements maintain crucial regulatory functions. Although the normal function of the *Cdx2* +7 kb element, if any, is not clear, binding by FoxA factors appears crucial for cryptic activation. The stringency with which FoxA is held in check is revealed by there being two nearby, repressive factor binding sites for Rfx and NHR-II.

Given that the genome appears to contain an abundance of cryptic regulatory elements [69], we suggest that our approach to investigating factor occupancy at silent genes will reveal the potential for other genetic programming transitions that are rare but can contribute to the basis for devastating human diseases.

## Materials and Methods

### Ethics statement

All work with animals for this study was performed in accordance with an approved IACUC protocol and relevant national guidelines. No animal survival studies were performed.

### Tiled array fabrication

Selected 210 genes of interest, and included 30 kb upstream and 10 kb downstream of the gene boundaries bases on UCSC Mouse Genome Browser, March 2005 build. Custom microarray containing 50mer probes with 24 bp overlap. Repetitive elements were masked. The final array contained 380,000 features. (Nimblegen). A list of the gene regions probed is provided in Table S1.

### Chromatin immunoprecipitation

Mouse livers from C3H strain, were crosslinked by perfusion through the portal vein and nuclei were isolated as described in [29]. Nuclei were resuspended in liver sonication buffer (50 mM Tris, 2 mM EDTA, 0.5% N-laurylsarcosine, 50 mM PMSF, protease inhibitors) held on ice for 5 min, then sheared in a bath sonicator (Diagenode Bioruptor) for 10 min with 30 sec ON/OFF intervals. RNAse was added to 40 ng/ml and chromatin was dialyzed to TE overnight 4°C, DNA was quantitated, and adjusted to 50 µg per ChIP reaction in 1X RIPA buffer. Immune complexes formed overnight at 4°C, and were recovered with 40 µl 50% pre-cleared protein A beads for 2 hr at 4°C. Beads were washed 6 times with high salt RIPA and eluted twice with 100 µl elution buffer (1% SDS, 0.1 M sodium bicarbonate) at 42°C. Protein-DNA crosslinks were reversed overnight at 68°C, and DNA was recovered by phenol-chloroform extraction followed by ethanol precipitation and resuspension in dH_2_0. For locus specific ChIP, enrichment was quantitated by standard curve analysis using SYBR Green QPCR (iCycler, BIO-RAD). A list of oligonucleotides used for ChIP-qPCR is provided in Table S3. For ChIP-chip analysis three competitive hybridizations were performed (FoxA2 vs IgG, FoxA2 vs input DNA, IgG vs input DNA). LM-PCR and DNA hybridization were performed per Nimblegen protocol. For cultured cells, ChIP was performed as described by Odom et al 2004. Sonication and immunoprecipitation performed as described for liver. Antibodies: FoxA2 (Upstate 07-633), Pol II phospho serine5 (Covance MMS-134R), IgG (Upstate), rabbit serum (Sigma), FoxA1 [29] and (Chemicon AB4124), Rfx1 (Santa Cruz sc-48809).

### Bioinformatics methods

To detect FoxA2 occupied regions or ChIP regions, we apply sliding-window method adapted from MAT [30] to the log-ratio intensities. For each window of a specific size *w*, a score 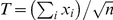 is calculated, where *x_i_* is the intensity of each probe in the window, and *n* is the number of probes in the window. The *T*-scores follow symmetric and asymptotically a normal distribution, therefore, the negative T-scores were used to estimate the empirical null distribution and *p*-values. Significant windows that are nearby each other, with a distance smaller than a specified gap size *g*, were merged, and the best T-score among them and the associated p-value were reported. We optimized the three parameters, window size *w* and gap size *g*, and p-value *p*, using a small subset of positive and negative controls. The reported results were obtained using *w* = 400 nt, *g* = 100 nt, and *p* = 0.0001. A region is called significant if *p*<0.0001 in the hybridization of either FoxA2 vs. IgG or FoxA2 vs. input DNA. We estimate an FDR of 16%, using the control hybridization of IgG vs. input DNA. To identify the motifs significantly enriched in the ChIP regions, we use 500-nt sequences centered in each ChIP region, and upstream/downstream sequences of the same size as background. We scan all 673 matrices of vertebrate transcription factors from TRANSFAC (Matys 2003) and JASPAR [70], by comparison occurrences of each matrix in ChIP and background datasets. The significance of enrichment in the ChIP regions was evaluated by a Binomial distribution, and the threshold of motif scores was optimized to maximize the enrichment. This analysis was performed using the motifclass program in the CREAD package (http://rulai.cshl.edu/cread/).

### Gene expression analysis

RNA was isolated using TRIzol reagent (Invitrogen) or RNeasy kit (QIAgen). 1 µg of RNA was converted to cDNA using iScript cDNA Synthesis kit (BIO-RAD.) and gene expression assessed by SYBR green QPCR using iCycler (BIO-RAD) or by TAQman low density arrays on an PRISM machine (Applied Biosystems.) Gene expression determined by the delta Ct method using HPRT as a reference. A list of Taqman probe IDs is provided in Table S4.

### Luciferase assays

Enhancer elements were PCR amplified and TOPO TA cloned (Invitrogen). Binding site mutations were introduced by overlap-extension PCR and constructs were sequence verified. Enhancer elements were subcloned into pGL3-promoter vector (Promega) downstream of the luciferase reporter gene. Cells were cultured in 6-well dishes and co-transfected with 1250 ng pGL3 vector and 125 ng pRL-CMV with Fugene reagent (Invitrogen). 72-hrs after transfection cells were lysed and luciferase activity was measured with the Dual-luciferase kit (Promega) per manufacturer's instructions.

### Histopathology and immunohistochemistry

We studied a tissue microarray containing 44 one millimeter cores from 22 anonymized esophageal mucosa samples, including 5 normal mucosa samples, from the gastroesophageal junction and 17 Barrett's esophagus lesions (11 Barrett's without dysplasia and 6 Barrett's with dysplasia). In addition, a total of twenty esophageal well differentiated invasive adenocarcinoma samples from the archives of FCCC were used. Paraffin sections were dewaxed using xylenes and hydrated using a series of ethanol. No antigen retrieval methods were necessary. Endogenous peroxidases were quenched with a short treatment of 1% hydrogen peroxide. Sections were incubated overnight with primary antibodies, washed the next day with PBS, incubated with biotinylated secondary antibodies (Vector Labs), incubated with Vecta Elite ABC kit (Vector Labs), developed with a DAB kit (Vector Labs) and counterstained with hematoxylin. Specimens were documented photographically using a Nikon Optiphot microscope, equipped with an Optronics CCD camera. Antibody against Rfx1 (Santa Cruz sc-10652) was used at a dilution of 1/50. Negative controls were incubated in the absence of primary antibodies as well as using a specific blocking peptide (Santa Cruz sc-10652 P) previously incubated together with the primary antibody at a concentration ten times higher than the latter. The percent of positively stained nuclei in normal and abnormal esophageal epithelium was determined by counting directly a total of 300–600 cells per sample (in at least three random fields per sample) using a micrometric eyepiece grid at a magnification of 400X.

### siRNA experiments

Smart pools (Dharmacon control siRNA D-001206-14-20; Rfx1 M-058841-01-0020) were added to cells in 6 cm dishes at 50 nM concentration using RNAifect (QIAGEN) per manufacturer's instruction. Cells were harvested for protein and RNA analysis 3 days post-transfection.

### Accession codes

Microarray data has been deposited to the NCBI GEO database.

## Supporting Information

Figure S1 (A) Pie chart representing the primary tissue of expression for the 210 selected genes on tiling array. (B) Schematic of tiling for gene regions on the microarray. Each tiled locus includes the gene coding region and flanking 30 kb upstream and 10 kb downstream sequence tiled at a density of one 50-nucleotide probe every 24 bp. In aggregate the array covers 14 Mb of the mouse genome. (C) Representative gel of chromatin shearing. (D) Comparison of FoxA2 ChIP to known sites in liver versus kidney, the latter being as a negative control that lacks FoxA2, showing the specificity of the FoxA2 antibody for FoxA2 antigen. (E) Bioanalyzer (Agilent) pseudo gel of DNA following LM-PCR, depicts the retention of small size distribution after amplification.
(TIF)

Figure S2 (A–C) Specificity of FoxA2 ChIP-chip. Region scores by sliding window analysis for ChIP-chip data (solid line) compared to randomized data (dotted line) for the three competitive hybridizations performed. Comparisons of FoxA2 with either IgG (A) or input DNA (B) using an empirical *p*-value of 0.0001 yielded ChIP-chip enrichment scores that were significantly above those expected from normally distributed data (red arrow). By contrast, the enrichment scores of the IgG ChIP vs input DNA comparison fit normal distributions very well, suggesting no significant ChIP regions (C). (D, E) FoxA2 ChIP validation of regions selected by ChIP-chip analysis. Site-specific ChIP-qPCR was performed from 4 mouse livers, and average signals greater than 2-fold enrichment over background were considered positive. (F) Pictogram of TRANSFAC FoxA motif (top) and that of *in vivo* FoxA binding motif determined by *de novo* motif analysis using 193 FoxA2 targets (bottom). (G) FoxA2 bound regions have a high degree of sequence conservation. A comparison of evolutionary conservation was performed between the sequences flanking the 193 ChIP-chip defined FoxA site (solid line) and sequences flanking the 2783 unbound predicted FoxA2 sites present in the intervals covered by the endoderm array (dotted line). A 2 kb region was extracted for each locus, and the PhastCons scores were averaged for each set of regions [71].
(TIF)

Figure S3 (A) Gene expression of 86 FoxA targets in liver, expressed as fraction of maximal expression. Weakly expressed and silent genes were binned as genes less than 1/10^4^ of albumin expression. Under this classification active FoxA target genes included A*lb1, TTR, FoxA2, Gata4, Xbp1, Prox1,* and *Hex*; whereas silent target genes included *AFP, Cdx2, SftpB,* and *Sox17*. (B) Gene activity expressed as function of distance between FoxA site and TSS showed that proximal FoxA binding was associated with gene activity.
(TIF)

Figure S4 Serial sections of Rfx1 IHC in a Barrett's esophagus in the presence of Rfx1 antibody (A), or Rfx1 antibody previously incubated with blocking peptide (1 to 10) (B). In (A) arrows indicate Rfx1 positive nuclei in the epithelium; arrowheads indicate Rfx1 positive nuclei in the stroma. Matched cells lacking Rfx1 positive stain are indicated in (B). Epithelial layer indicated by “E”; stromal cells indicated by “S”.
(TIF)

Figure S5 Loss of Rfx1 expression in esophageal adenocarcinoma. Western blots with Rfx1 antibody and GAPDH control, using extracts from H520 lung cancer cell line as a positive control and 6 anonymized samples of esophageal adenocarcinoma. Numbers to right indicate relative molecular masses of standards (not shown) in kD.
(JPG)

Table S1 Endoderm Array Gene Loci.
(DOCX)

Table S2 Liver FoxA Binding Sites.
(DOCX)

Table S3 Primers for ChIP-qPCR.
(DOCX)

Table S4 Taqman Probe Ids.
(DOCX)
