# Supplementary figures and images for: Study of FoxA Pioneer Factor at Silent Genes Reveals Rfx-Repressed Enhancer at Cdx2 and a Potential Indicator of Esophageal Adenocarcinoma Development
Source: PLoS Genet. 2011 Sep 15;7(9):e1002277. doi: 10.1371/journal.pgen.1002277 (PMC3174211; doi:10.1371/journal.pgen.1002277)

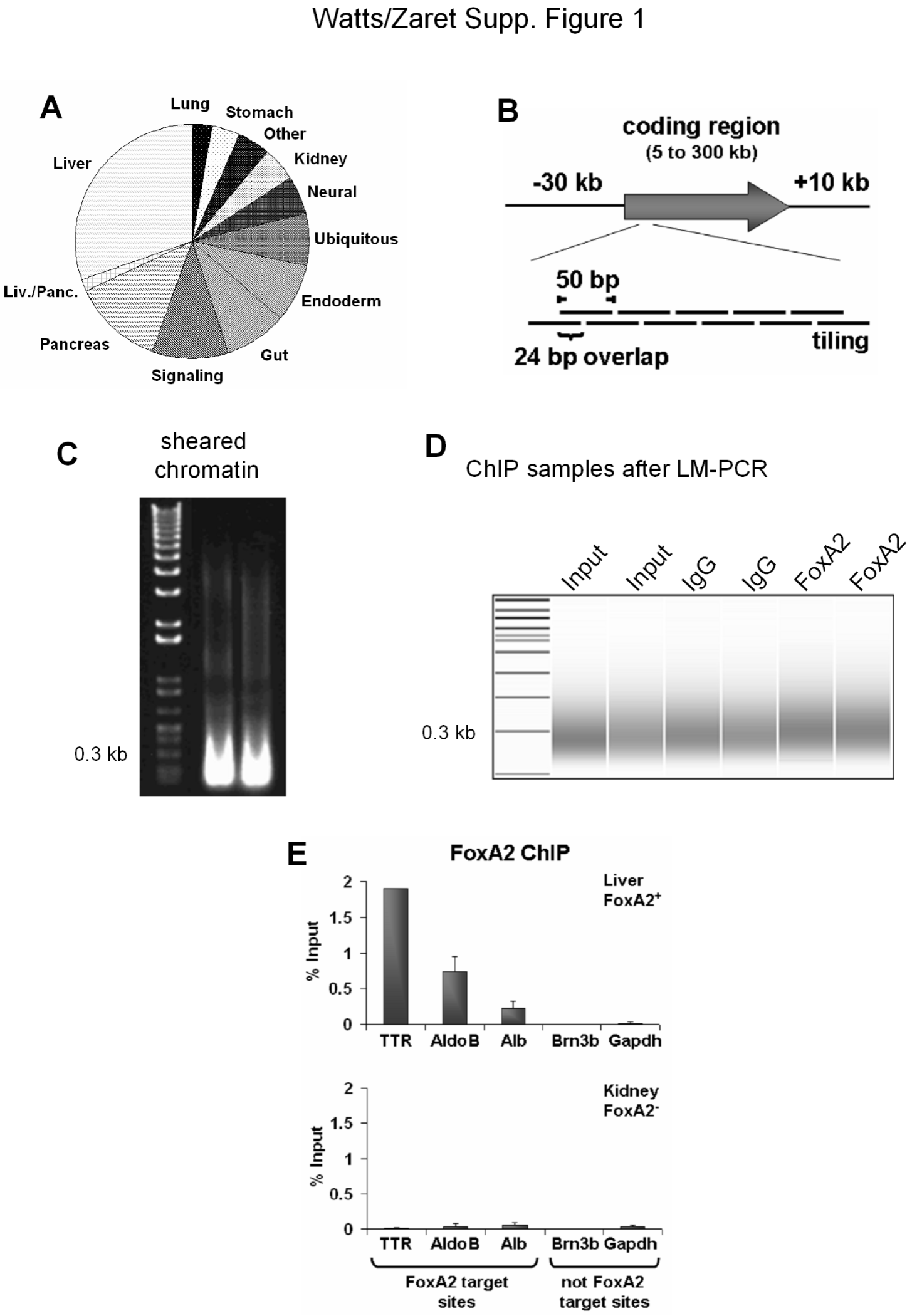

Supplement: Figure S1 — (A) Pie chart representing the primary tissue of expression for the 210 selected genes on tiling array. (B) Schematic of tiling for gene regions on the microarray. Each tiled locus includes the gene coding region and flanking 30 kb upstream and 10 kb downstream sequence tiled at a density of one 50-nucleotide probe every 24 bp. In aggregate the array covers 14 Mb of the mouse genome. (C) Representative gel of chromatin shearing. (D) Comparison of FoxA2 ChIP to known sites in liver versus kidney, the latter being as a negative control that lacks FoxA2, showing the specificity of the FoxA2 antibody for FoxA2 antigen. (E) Bioanalyzer (Agilent) pseudo gel of DNA following LM-PCR, depicts the retention of small size distribution after amplification. (TIF) [file pgen.1002277.s001.tif]

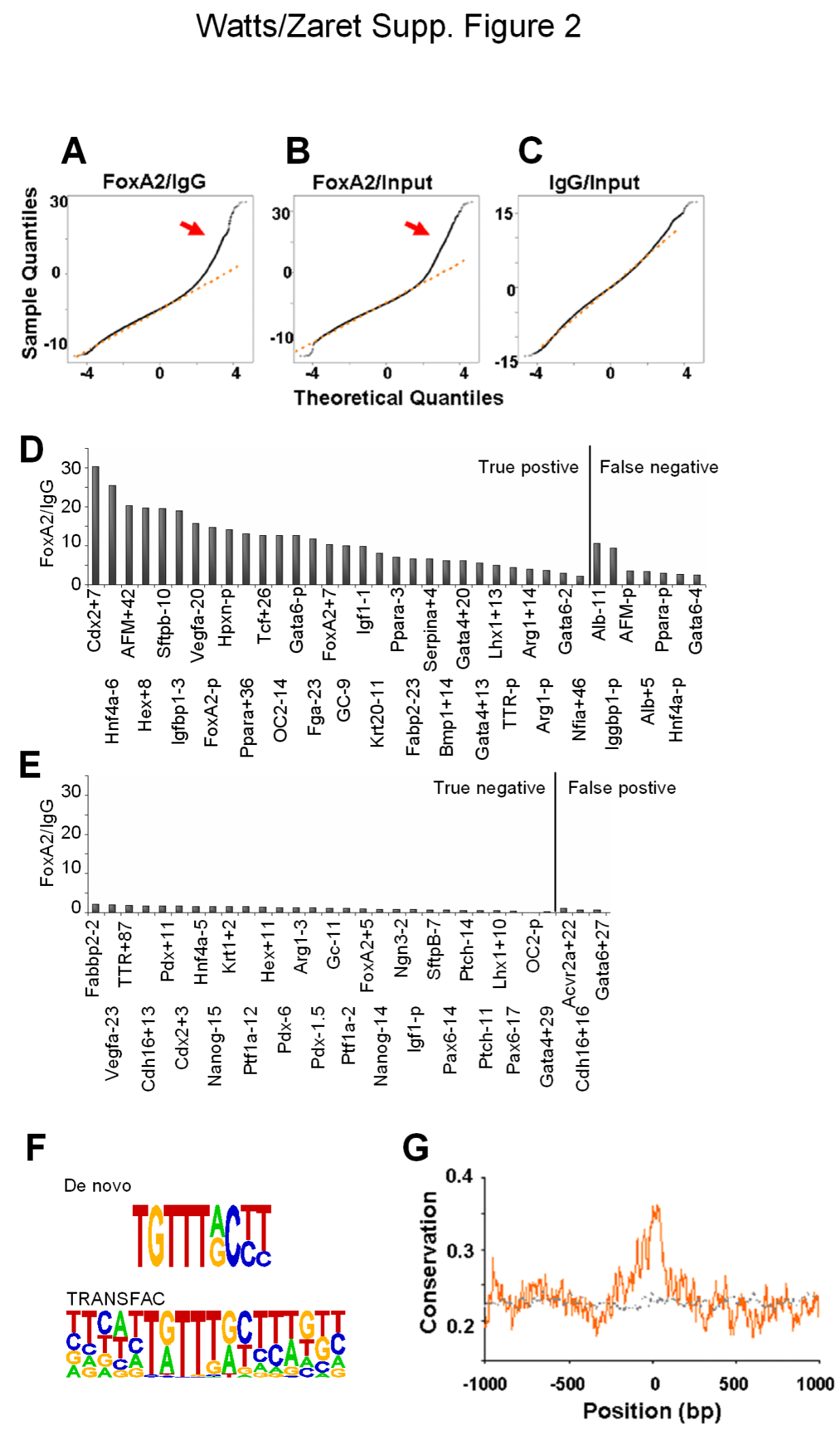

Supplement: Figure S2 — (A–C) Specificity of FoxA2 ChIP-chip. Region scores by sliding window analysis for ChIP-chip data (solid line) compared to randomized data (dotted line) for the three competitive hybridizations performed. Comparisons of FoxA2 with either IgG (A) or input DNA (B) using an empirical p-value of 0.0001 yielded ChIP-chip enrichment scores that were significantly above those expected from normally distributed data (red arrow). By contrast, the enrichment scores of the IgG ChIP vs input DNA comparison fit normal distributions very well, suggesting no significant ChIP regions (C). (D, E) FoxA2 ChIP validation of regions selected by ChIP-chip analysis. Site-specific ChIP-qPCR was performed from 4 mouse livers, and average signals greater than 2-fold enrichment over background were considered positive. (F) Pictogram of TRANSFAC FoxA motif (top) and that of in vivo FoxA binding motif determined by de novo motif analysis using 193 FoxA2 targets (bottom). (G) FoxA2 bound regions have a high degree of sequence conservation. A comparison of evolutionary conservation was performed between the sequences flanking the 193 ChIP-chip defined FoxA site (solid line) and sequences flanking the 2783 unbound predicted FoxA2 sites present in the intervals covered by the endoderm array (dotted line). A 2 kb region was extracted for each locus, and the PhastCons scores were averaged for each set of regions [71]. (TIF) [file pgen.1002277.s002.tif]

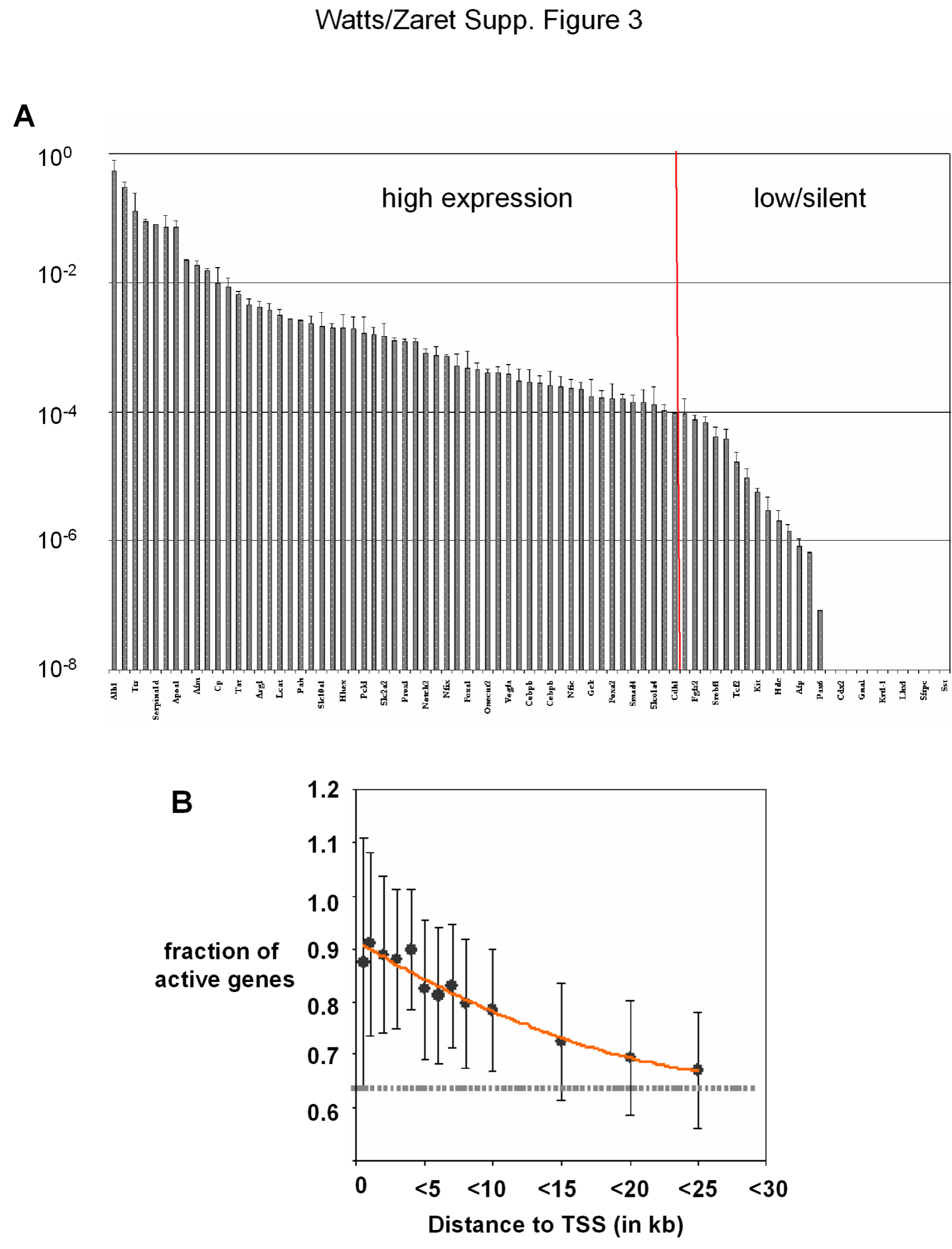

Supplement: Figure S3 — (A) Gene expression of 86 FoxA targets in liver, expressed as fraction of maximal expression. Weakly expressed and silent genes were binned as genes less than 1/104 of albumin expression. Under this classification active FoxA target genes included Alb1, TTR, FoxA2, Gata4, Xbp1, Prox1, and Hex; whereas silent target genes included AFP, Cdx2, SftpB, and Sox17. (B) Gene activity expressed as function of distance between FoxA site and TSS showed that proximal FoxA binding was associated with gene activity. (TIF) [file pgen.1002277.s003.tif]

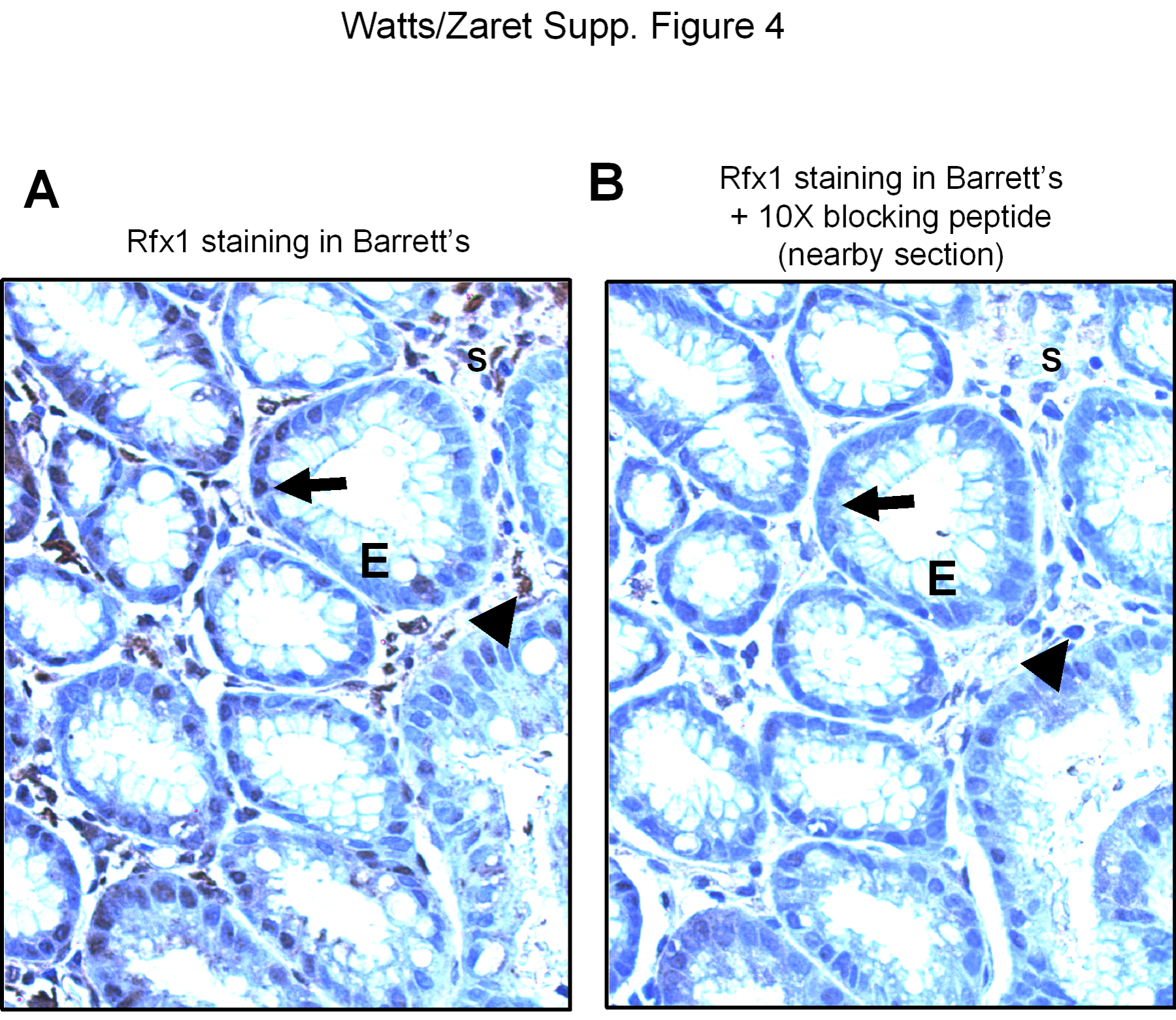

Supplement: Figure S4 — Serial sections of Rfx1 IHC in a Barrett's esophagus in the presence of Rfx1 antibody (A), or Rfx1 antibody previously incubated with blocking peptide (1 to 10) (B). In (A) arrows indicate Rfx1 positive nuclei in the epithelium; arrowheads indicate Rfx1 positive nuclei in the stroma. Matched cells lacking Rfx1 positive stain are indicated in (B). Epithelial layer indicated by “E”; stromal cells indicated by “S”. (TIF) [file pgen.1002277.s004.tif]

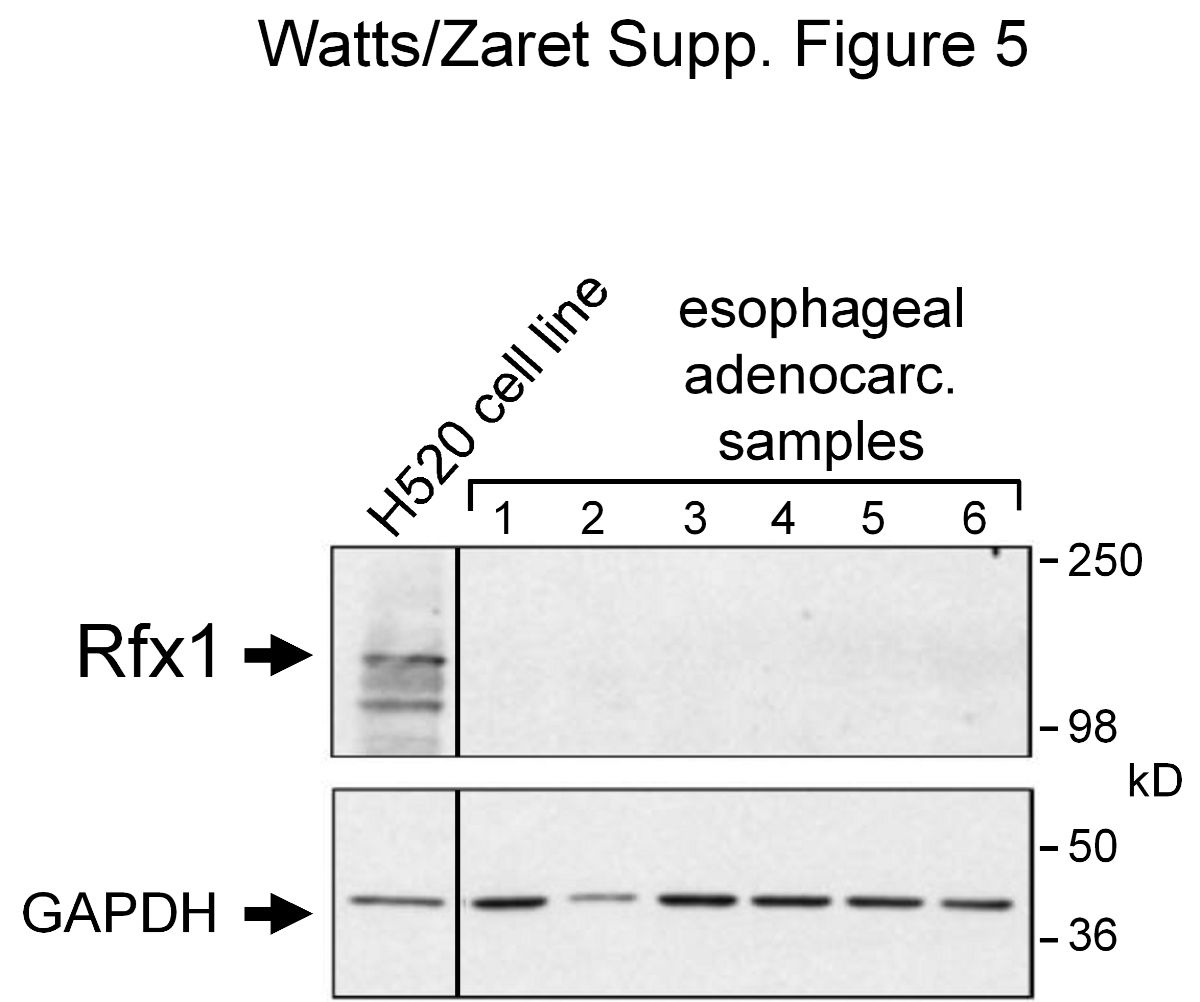

Supplement: Figure S5 — Loss of Rfx1 expression in esophageal adenocarcinoma. Western blots with Rfx1 antibody and GAPDH control, using extracts from H520 lung cancer cell line as a positive control and 6 anonymized samples of esophageal adenocarcinoma. Numbers to right indicate relative molecular masses of standards (not shown) in kD. (JPG) [file pgen.1002277.s005.jpg]
