# Supplementary material for: Study of FoxA Pioneer Factor at Silent Genes Reveals Rfx-Repressed Enhancer at Cdx2 and a Potential Indicator of Esophageal Adenocarcinoma Development
Source: PLoS Genet. 2011 Sep 15;7(9):e1002277. doi: 10.1371/journal.pgen.1002277 (PMC3174211; doi:10.1371/journal.pgen.1002277)
Supplement: Table S1 — Endoderm Array Gene Loci. (DOCX) [file pgen.1002277.s006.docx]

| **Supplemental Table 1: Endoderm Array Gene Loci** | | | | |  |  |  |
| --- | --- | --- | --- | --- | --- | --- | --- |
|  |  |  |  |  |  |  |  |
| **RefSeq DNA** | **Gene Symbol** | **Chr** | **Prox Gene Boundary** | **Distal Gene Boundary** | **Orient.** | **Gene size** | **Size of Locus** |
| [NM_007393](http://genome-www5.stanford.edu/cgi-bin/SMD/source/sourceResult?option=Number&criteria=NM_007393&choice=Gene) | Actb | 5 | 141,967,352 | 141,970,959 | -1 | 3,607 | 43607 |
| [NM_007396](http://genome-www5.stanford.edu/cgi-bin/SMD/source/sourceResult?option=Number&criteria=NM_007396&choice=Gene) | Acvr2a | 2 | 48746319 | 48834326 | 1 | 88,007 | 128007 |
| [NM_007397](http://genome-www5.stanford.edu/cgi-bin/SMD/source/sourceResult?option=Number&criteria=NM_007397&choice=Gene) | Acvr2b | 9 | 119,396,762 | 119,427,777 | 1 | 31,015 | 71015 |
| [NM_007398](http://genome-www5.stanford.edu/cgi-bin/SMD/source/sourceResult?option=Number&criteria=NM_007398&choice=Gene) | ADA | 2 | 163,183,553 | 163,207,147 | -1 | 23,594 | 63594 |
| [NM_145146](http://genome-www5.stanford.edu/cgi-bin/SMD/source/sourceResult?option=Number&criteria=NM_145146&choice=Gene) | AFM | 5 | 89,816,391 | 89,851,133 | 1 | 34,742 | 74742 |
| [NM_007423](http://genome-www5.stanford.edu/cgi-bin/SMD/source/sourceResult?option=Number&criteria=NM_007423&choice=Gene) | AFP | 5 | 89,788,165 | 89,806,335 | 1 | 18,170 | 58170 |
| [NM_009654](http://genome-www5.stanford.edu/cgi-bin/SMD/source/sourceResult?option=Number&criteria=NM_009654&choice=Gene) | Alb | 5 | 89,758,329 | 89,774,029 | 1 | 15,700 | 55700 |
|  | Alb-AFM | 5 | 89758329 | 89851133 | NA | 92,804 | 152804 |
| [NM_144903](http://genome-www5.stanford.edu/cgi-bin/SMD/source/sourceResult?option=Number&criteria=NM_144903&choice=Gene) | AldoB | 4 | 49,451,871 | 49,465,358 | -1 | 13,487 | 53487 |
| [NM_009669](http://genome-www5.stanford.edu/cgi-bin/SMD/source/sourceResult?option=Number&criteria=NM_009669&choice=Gene) | amylase2 | 3 | 113,188,371 | 113,197,828 | -1 | 9,457 | 49457 |
| [NM_013913](http://genome-www5.stanford.edu/cgi-bin/SMD/source/sourceResult?option=Number&criteria=NM_013913&choice=Gene) | Angptl3 | 4 | 98,006,061 | 98,013,098 | 1 | 7,037 | 47037 |
| [NM_009692](http://genome-www5.stanford.edu/cgi-bin/SMD/source/sourceResult?option=Number&criteria=NM_009692&choice=Gene) | ApoA1 | 9 | 46,241,962 | 46,243,717 | 1 | 1,755 | 41755 |
| [NM_013474](http://genome-www5.stanford.edu/cgi-bin/SMD/source/sourceResult?option=Number&criteria=NM_013474&choice=Gene) | Apoa2 | 1 | 171,153,928 | 171,155,210 | 1 | 1,282 | 41282 |
| [NM_007468](http://genome-www5.stanford.edu/cgi-bin/SMD/source/sourceResult?option=Number&criteria=NM_007468&choice=Gene) | Apoa4 | 9 | 46,254,406 | 46,256,542 | 1 | 2,136 | 42136 |
| [BC028880](http://www.ncbi.nlm.nih.gov/entrez/query.fcgi?cmd=Search&db=Nucleotide&term=BC028880&doptcmdl=GenBank&tool=genome.ucsc.edu) | apob | 12 | 7,223,049 | 7,251,330 | 1 | 28,281 | 68281 |
| [NM_023114](http://genome-www5.stanford.edu/cgi-bin/SMD/source/sourceResult?option=Number&criteria=NM_023114&choice=Gene) | Apoc3 | 9 | 46246303 | 46248550 | -1 | 2,247 | 42247 |
| [NM_009696](http://genome-www5.stanford.edu/cgi-bin/SMD/source/sourceResult?option=Number&criteria=NM_009696&choice=Gene) | ApoE | 7 | 16,565,517 | 16,568,277 | -1 | 2,760 | 42760 |
| [NM_007482](http://genome-www5.stanford.edu/cgi-bin/SMD/source/sourceResult?option=Number&criteria=NM_007482&choice=Gene) | Arg1 | 10 | 24,891,463 | 24,903,677 | -1 | 12,214 | 52214 |
| [NM_007492](http://genome-www5.stanford.edu/cgi-bin/SMD/source/sourceResult?option=Number&criteria=NM_007492&choice=Gene) | Arx | X | 87,947,304 | 87,959,012 | 1 | 11,708 | 51708 |
| [NM_009724](http://genome-www5.stanford.edu/cgi-bin/SMD/source/sourceResult?option=Number&criteria=NM_009724&choice=Gene) | Atp4b | 8 | 12,764,848 | 12,775,461 | -1 | 10,613 | 50613 |
| [NM_007526](http://genome-www5.stanford.edu/cgi-bin/SMD/source/sourceResult?option=Number&criteria=NM_007526&choice=Gene) | Barx1 | 13 | 48,261,095 | 48,264,510 | 1 | 3,415 | 43415 |
| [NM_009309](http://genome-www5.stanford.edu/cgi-bin/SMD/source/sourceResult?option=Number&criteria=NM_009309&choice=Gene) | Brachyury | 17 | 13,232,430 | 13,240,504 | 1 | 8,074 | 48074 |
| [NM_138944](http://genome-www5.stanford.edu/cgi-bin/SMD/source/sourceResult?option=Number&criteria=NM_138944&choice=Gene) | Brn3b | 8 | 77,646,129 | 77,649,773 | -1 | 3,644 | 43644 |
| [NM_007678](http://genome-www5.stanford.edu/cgi-bin/SMD/source/sourceResult?option=Number&criteria=NM_007678&choice=Gene) | C/EBPa | 7 | 30,276,207 | 30,277,488 | 1 | 1,281 | 41281 |
| [NM_009883](http://genome-www5.stanford.edu/cgi-bin/SMD/source/sourceResult?option=Number&criteria=NM_009883&choice=Gene) | C/EBPb | 2 | 167,197,648 | 167,199,153 | 1 | 1,505 | 41505 |
| [NM_009803](http://genome-www5.stanford.edu/cgi-bin/SMD/source/sourceResult?option=Number&criteria=NM_009803&choice=Gene) | CAR | 1 | 171,142,841 | 171,147,684 | 1 | 4,843 | 44843 |
| [NM_011681](http://genome-www5.stanford.edu/cgi-bin/SMD/source/sourceResult?option=Number&criteria=NM_011681&choice=Gene) | CC10 | 19 | 8,280,190 | 8,284,461 | -1 | 4,271 | 44271 |
| [NM_031161](http://genome-www5.stanford.edu/cgi-bin/SMD/source/sourceResult?option=Number&criteria=NM_031161&choice=Gene) | CCK | 9 | 121,506,267 | 121,512,103 | -1 | 5,836 | 45836 |
| [NM_009864](http://genome-www5.stanford.edu/cgi-bin/SMD/source/sourceResult?option=Number&criteria=NM_009864&choice=Gene) | cdh1 | 8 | 105,899,006 | 105,965,884 | 1 | 66,878 | 106878 |
| [NM_007663](http://genome-www5.stanford.edu/cgi-bin/SMD/source/sourceResult?option=Number&criteria=NM_007663&choice=Gene) | Cdh16 | 8 | 103,907,283 | 103,917,397 | -1 | 10,114 | 50114 |
| [NM_009880](http://genome-www5.stanford.edu/cgi-bin/SMD/source/sourceResult?option=Number&criteria=NM_009880&choice=Gene) | cdx1 | 18 | 61244191 | 61261526 | -1 | 17,335 | 57335 |
| [NM_007673](http://genome-www5.stanford.edu/cgi-bin/SMD/source/sourceResult?option=Number&criteria=NM_007673&choice=Gene) | cdx2 | 5 | 146,192,396 | 146,198,745 | -1 | 6,349 |  |
| [NM_009887](http://genome-www5.stanford.edu/cgi-bin/SMD/source/sourceResult?option=Number&criteria=NM_009887&choice=Gene) | cer1 | 4 | 81,867,943 | 81,871,344 | -1 | 3,401 | 43401 |
| [NM_009893](http://genome-www5.stanford.edu/cgi-bin/SMD/source/sourceResult?option=Number&criteria=NM_009893&choice=Gene) | chrd | 16 | 19,504,234 | 19,513,490 | 1 | 9,256 | 49256 |
| [NM_013491](http://genome-www5.stanford.edu/cgi-bin/SMD/source/sourceResult?option=Number&criteria=NM_013491&choice=Gene) | clcn1 | 6 | 42,430,565 | 42,458,536 | 1 | 27,971 | 67971 |
| [NM_009900](http://genome-www5.stanford.edu/cgi-bin/SMD/source/sourceResult?option=Number&criteria=NM_009900&choice=Gene) | clcn2 | 16 | 19,475,107 | 19,488,387 | -1 | 13,280 | 53280 |
| [NM_018777](http://genome-www5.stanford.edu/cgi-bin/SMD/source/sourceResult?option=Number&criteria=NM_018777&choice=Gene) | Cldn6 | 17 | 21,483,245 | 21,483,903 | 1 | 658 | 40658 |
| [NM_007752](http://genome-www5.stanford.edu/cgi-bin/SMD/source/sourceResult?option=Number&criteria=NM_007752&choice=Gene) | Cp | 3 | 19,298,453 | 19,333,865 | 1 | 35,412 | 75412 |
| [BC093513](http://www.ncbi.nlm.nih.gov/entrez/query.fcgi?cmd=Search&db=Nucleotide&term=BC093513&doptcmdl=GenBank&tool=genome.ucsc.edu) | cps1 | 1 | 67,466,664 | 67,523,556 | 1 | 56,892 | 96892 |
| [NM_013496](http://genome-www5.stanford.edu/cgi-bin/SMD/source/sourceResult?option=Number&criteria=NM_013496&choice=Gene) | Crabp1 | 9 | 54,882,658 | 54,891,163 | 1 | 8,505 | 48505 |
| [NM_145365](http://genome-www5.stanford.edu/cgi-bin/SMD/source/sourceResult?option=Number&criteria=NM_145365&choice=Gene) | CREB-H | 10 | 81,218,000 | 81,232,761 | -1 | 14,761 | 54761 |
| [NM_007768](http://genome-www5.stanford.edu/cgi-bin/SMD/source/sourceResult?option=Number&criteria=NM_007768&choice=Gene) | CRP | 1 | 172,626,985 | 172,628,880 | 1 | 1,895 | 41895 |
| [NM_007614](http://genome-www5.stanford.edu/cgi-bin/SMD/source/sourceResult?option=Number&criteria=NM_007614&choice=Gene) | ctnnb1 | 9 | 120,945,869 | 120,972,901 | 1 | 27,032 | 67032 |
| [NM_009911](http://genome-www5.stanford.edu/cgi-bin/SMD/source/sourceResult?option=Number&criteria=NM_009911&choice=Gene) | cxcr4 | 1 | 128,431,065 | 128,435,145 | -1 | 4,080 | 44080 |
| [NM_007824](http://genome-www5.stanford.edu/cgi-bin/SMD/source/sourceResult?option=Number&criteria=NM_007824&choice=Gene) | Cyp7a1 | 4 | 6,192,758 | 6,202,778 | -1 | 10,020 | 50020 |
| [NM_016974](http://genome-www5.stanford.edu/cgi-bin/SMD/source/sourceResult?option=Number&criteria=NM_016974&choice=Gene) | Dbp | 7 | 39,788,555 | 39,793,475 | 1 | 4,920 | 44920 |
| [NM_016672](http://genome-www5.stanford.edu/cgi-bin/SMD/source/sourceResult?option=Number&criteria=NM_016672&choice=Gene) | DDC | 11 | 11,709,235 | 11,775,443 | -1 | 66,208 | 106208 |
| [NM_145157](http://genome-www5.stanford.edu/cgi-bin/SMD/source/sourceResult?option=Number&criteria=NM_145157&choice=Gene) | Defb19 | 2 | 152,033,023 | 152,037,249 | -1 | 4,226 | 44226 |
| [NM_010051](http://genome-www5.stanford.edu/cgi-bin/SMD/source/sourceResult?option=Number&criteria=NM_010051&choice=Gene) | Dkk1 | 19 | 29,797,795 | 29,801,407 | -1 | 3,612 | 43612 |
| [NM_010052](http://genome-www5.stanford.edu/cgi-bin/SMD/source/sourceResult?option=Number&criteria=NM_010052&choice=Gene) | Dlk1 | 12 | 104,931,430 | 104,938,899 | 1 | 7,469 | 47469 |
| [NM_010074](http://genome-www5.stanford.edu/cgi-bin/SMD/source/sourceResult?option=Number&criteria=NM_010074&choice=Gene) | Dpp4 | 2 | 62,187,140 | 62,267,483 | -1 | 80,343 | 120343 |
| [NM_010110](http://genome-www5.stanford.edu/cgi-bin/SMD/source/sourceResult?option=Number&criteria=NM_010110&choice=Gene) | Efnb1 | X | 93737491 | 93750353 | 1 | 12862 | 52862 |
| [NM_010111](http://genome-www5.stanford.edu/cgi-bin/SMD/source/sourceResult?option=Number&criteria=NM_010111&choice=Gene) | Efnb2 | 8 | 7,983,291 | 8,026,620 | -1 | 43,329 | 83329 |
| [NM_007921](http://genome-www5.stanford.edu/cgi-bin/SMD/source/sourceResult?option=Number&criteria=NM_007921&choice=Gene) | Elf3 | 1 | 135,104,074 | 135,108,932 | -1 | 4,858 | 44858 |
| [NM_021099](http://genome-www5.stanford.edu/cgi-bin/SMD/source/sourceResult?option=Number&criteria=NM_021099&choice=Gene) | ENSMUST00000005815 | 5 | 74,409,245 | 74,490,902 | 1 | 81,657 | 121657 |
| [NM_010136](http://genome-www5.stanford.edu/cgi-bin/SMD/source/sourceResult?option=Number&criteria=NM_010136&choice=Gene) | Eomes | 9 | 118,472,585 | 118,480,374 | 1 | 7,789 | 47789 |
| [NM_010142](http://genome-www5.stanford.edu/cgi-bin/SMD/source/sourceResult?option=Number&criteria=NM_010142&choice=Gene) | Ephb2 | 4 | 135,534,871 | 135,717,193 | -1 | 182,322 | 222322 |
| [NM_010143](http://genome-www5.stanford.edu/cgi-bin/SMD/source/sourceResult?option=Number&criteria=NM_010143&choice=Gene) | Ephb3 | 16 | 19,975,855 | 19,994,364 | 1 | 18,509 | 58509 |
| [NM_017399](http://genome-www5.stanford.edu/cgi-bin/SMD/source/sourceResult?option=Number&criteria=NM_017399&choice=Gene) | Fabp1 | 6 | 71,531,814 | 71,536,949 | 1 | 5,135 | 45135 |
| [NM_007980](http://genome-www5.stanford.edu/cgi-bin/SMD/source/sourceResult?option=Number&criteria=NM_007980&choice=Gene) | Fabp2 | 3 | 121,686,438 | 121,690,554 | 1 | 4,116 | 44116 |
| [NM_010196](http://genome-www5.stanford.edu/cgi-bin/SMD/source/sourceResult?option=Number&criteria=NM_010196&choice=Gene) | Fga | 3 | 82,755,131 | 82,761,238 | 1 | 6,107 | 46107 |
| [NM_010206](http://genome-www5.stanford.edu/cgi-bin/SMD/source/sourceResult?option=Number&criteria=NM_010206&choice=Gene) | Fgfr1 | 8 | 24,257,087 | 24,299,020 | 1 | 41,933 | 81933 |
| [NM_201601](http://genome-www5.stanford.edu/cgi-bin/SMD/source/sourceResult?option=Number&criteria=NM_201601&choice=Gene) | Fgfr2 | 7 | 124,236,726 | 124,389,453 | -1 | 152,727 | 192727 |
| [NM_008011](http://genome-www5.stanford.edu/cgi-bin/SMD/source/sourceResult?option=Number&criteria=NM_008011&choice=Gene) | Fgfr4 | 13 | 53,763,912 | 53,779,775 | 1 | 15,863 | 55863 |
| [NM_145594](http://genome-www5.stanford.edu/cgi-bin/SMD/source/sourceResult?option=Number&criteria=NM_145594&choice=Gene) | Fgl1 | 8 | 40,129,069 | 40,152,759 | -1 | 23,690 | 63690 |
| [NM_008259](http://genome-www5.stanford.edu/cgi-bin/SMD/source/sourceResult?option=Number&criteria=NM_008259&choice=Gene) | FoxA1 | 12 | 54,272,111 | 54,277,440 | -1 | 5,329 | 45329 |
| [NM_010446](http://genome-www5.stanford.edu/cgi-bin/SMD/source/sourceResult?option=Number&criteria=NM_010446&choice=Gene) | FoxA2 | 2 | 147,499,859 | 147,503,906 | -1 | 4,047 | 44047 |
| [NM_008260](http://genome-www5.stanford.edu/cgi-bin/SMD/source/sourceResult?option=Number&criteria=NM_008260&choice=Gene) | FoxA3 | 7 | 15,882,044 | 15,892,301 | -1 | 10,257 | 50257 |
| [NM_008592](http://genome-www5.stanford.edu/cgi-bin/SMD/source/sourceResult?option=Number&criteria=NM_008592&choice=Gene) | FoxC1 | 13 | 31,286,268 | 31,290,257 | 1 | 3,989 | 43989 |
| [NM_013519](http://genome-www5.stanford.edu/cgi-bin/SMD/source/sourceResult?option=Number&criteria=NM_013519&choice=Gene) | FoxC2 | 8 | 120,472,104 | 120,474,412 | 1 | 2,308 | 42308 |
| [NM_010426](http://genome-www5.stanford.edu/cgi-bin/SMD/source/sourceResult?option=Number&criteria=NM_010426&choice=Gene) | Foxf1a | 8 | 120,439,996 | 120,442,400 | 1 | 2,404 | 42404 |
| [NM_008021](http://genome-www5.stanford.edu/cgi-bin/SMD/source/sourceResult?option=Number&criteria=NM_008021&choice=Gene) | Foxm1 | 6 | 129,053,163 | 129,066,069 | 1 | 12,906 | 52906 |
| [NM_008061](http://genome-www5.stanford.edu/cgi-bin/SMD/source/sourceResult?option=Number&criteria=NM_008061&choice=Gene) | G6PC | 11 | 101,188,817 | 101,198,989 | 1 | 10,172 | 50172 |
| [NM_001001303](http://genome-www5.stanford.edu/cgi-bin/SMD/source/sourceResult?option=Number&criteria=NM_001001303&choice=Gene) | Gapdh | 6 | 125,818,147 | 125,821,886 | -1 | 3,739 | 43739 |
| [NM_010257](http://genome-www5.stanford.edu/cgi-bin/SMD/source/sourceResult?option=Number&criteria=NM_010257&choice=Gene) | gastrin | 11 | 100,155,494 | 100,158,084 | 1 | 2,590 | 42590 |
| [NM_008092](http://genome-www5.stanford.edu/cgi-bin/SMD/source/sourceResult?option=Number&criteria=NM_008092&choice=Gene) | Gata4 | 14 | 57,729,513 | 57,775,852 | -1 | 46,339 | 86339 |
| [NM_008093](http://genome-www5.stanford.edu/cgi-bin/SMD/source/sourceResult?option=Number&criteria=NM_008093&choice=Gene) | Gata5 | 2 | 180,042,091 | 180,051,632 | -1 | 9,541 | 49541 |
| [NM_010258](http://genome-www5.stanford.edu/cgi-bin/SMD/source/sourceResult?option=Number&criteria=NM_010258&choice=Gene) | Gata6 | 18 | 11,097,852 | 11,130,974 | 1 | 33,122 | 73122 |
| [NM_008096](http://genome-www5.stanford.edu/cgi-bin/SMD/source/sourceResult?option=Number&criteria=NM_008096&choice=Gene) | Gc | 5 | 88,714,420 | 88,756,682 | -1 | 42,262 | 82262 |
| [NM_008100](http://genome-www5.stanford.edu/cgi-bin/SMD/source/sourceResult?option=Number&criteria=NM_008100&choice=Gene) | gcg | 2 | 62,329,931 | 62,339,054 | -1 | 9,123 | 49123 |
| [NM_010292](http://genome-www5.stanford.edu/cgi-bin/SMD/source/sourceResult?option=Number&criteria=NM_010292&choice=Gene) | Gck | 11 | 5,795,610 | 5,844,387 | 1 | 48,777 | 88777 |
| [NM_021488](http://genome-www5.stanford.edu/cgi-bin/SMD/source/sourceResult?option=Number&criteria=NM_021488&choice=Gene) | ghrelin | 6 | 114,284,706 | 114,288,468 | -1 | 3,762 | 43762 |
| [NM_008118](http://genome-www5.stanford.edu/cgi-bin/SMD/source/sourceResult?option=Number&criteria=NM_008118&choice=Gene) | Gif | 19 | 10,944,054 | 10,959,943 | 1 | 15,889 | 55889 |
| [NM_011216](http://genome-www5.stanford.edu/cgi-bin/SMD/source/sourceResult?option=Number&criteria=NM_011216&choice=Gene) | Glepp1 | 6 | 138,044,285 | 138,255,223 | 1 | 210,938 | 250938 |
| [BC031171](http://www.ncbi.nlm.nih.gov/entrez/query.fcgi?cmd=Search&db=Nucleotide&term=BC031171&doptcmdl=GenBank&tool=genome.ucsc.edu) | Gli2 | 1 | 118,585,569 | 118,588,837 | -1 | 3,268 | 43268 |
| [NM_008130](http://genome-www5.stanford.edu/cgi-bin/SMD/source/sourceResult?option=Number&criteria=NM_008130&choice=Gene) | Gli3 | 13 | 14,911,831 | 15,175,565 | 1 | 263,734 | 303734 |
| [NM_008131](http://genome-www5.stanford.edu/cgi-bin/SMD/source/sourceResult?option=Number&criteria=NM_008131&choice=Gene) | Glu1 | 1 | 153,783,869 | 153,793,638 | 1 | 9,769 | 49769 |
| [NM_031197](http://genome-www5.stanford.edu/cgi-bin/SMD/source/sourceResult?option=Number&criteria=NM_031197&choice=Gene) | glut2 | 3 | 28,115,361 | 28,145,712 | 1 | 30,351 | 70351 |
| [NM_177137](http://genome-www5.stanford.edu/cgi-bin/SMD/source/sourceResult?option=Number&criteria=NM_177137&choice=Gene) | Gna1 | 18 | 67,317,670 | 67,453,587 | 1 | 135,917 | 175917 |
| [NM_027817](http://genome-www5.stanford.edu/cgi-bin/SMD/source/sourceResult?option=Number&criteria=NM_027817&choice=Gene) | grap | 11 | 61,378,964 | 61,398,420 | 1 | 19,456 | 59456 |
| [NM_010351](http://genome-www5.stanford.edu/cgi-bin/SMD/source/sourceResult?option=Number&criteria=NM_010351&choice=Gene) | Gsc | 12 | 99,915,345 | 99,917,373 | -1 | 2,028 | 42028 |
| [BC025150](http://www.ncbi.nlm.nih.gov/entrez/query.fcgi?cmd=Search&db=Nucleotide&term=BC025150&doptcmdl=GenBank&tool=genome.ucsc.edu) | H19 | 7 | 136,988,552 | 136,990,035 | 1 | 1,483 |  |
|  | H19-Igf2 | 7 |  |  | NA | 86,329 | 146329 |
| [NM_008230](http://genome-www5.stanford.edu/cgi-bin/SMD/source/sourceResult?option=Number&criteria=NM_008230&choice=Gene) | Hdc | 2 | 126,107,521 | 126,132,519 | -1 | 24,998 | 64998 |
| [NM_008235](http://genome-www5.stanford.edu/cgi-bin/SMD/source/sourceResult?option=Number&criteria=NM_008235&choice=Gene) | HEs1 | 16 | 28,870,268 | 28,872,833 | 1 | 2,565 | 42565 |
| [NM_008245](http://genome-www5.stanford.edu/cgi-bin/SMD/source/sourceResult?option=Number&criteria=NM_008245&choice=Gene) | Hex | 19 | 36,778,120 | 36,784,011 | 1 | 5,891 | 45891 |
| [NM_010427](http://genome-www5.stanford.edu/cgi-bin/SMD/source/sourceResult?option=Number&criteria=NM_010427&choice=Gene) | HGF | 5 | 14,975,130 | 15,041,018 | 1 | 65,888 | 105888 |
| [NM_019944](http://genome-www5.stanford.edu/cgi-bin/SMD/source/sourceResult?option=Number&criteria=NM_019944&choice=Gene) | Hlxb9 | 5 | 27923745 | 27928953 | -1 | 5,208 | 45208 |
| [NM_009327](http://genome-www5.stanford.edu/cgi-bin/SMD/source/sourceResult?option=Number&criteria=NM_009327&choice=Gene) | HNF1alpha | 5 | 114,057,988 | 114,080,665 | -1 | 22,677 | 62677 |
| [NM_009330](http://genome-www5.stanford.edu/cgi-bin/SMD/source/sourceResult?option=Number&criteria=NM_009330&choice=Gene) | HNF1beta | 11 | 83,579,041 | 83,633,337 | 1 | 54,296 | 94296 |
| [NM_008261](http://genome-www5.stanford.edu/cgi-bin/SMD/source/sourceResult?option=Number&criteria=NM_008261&choice=Gene) | Hnf4a | 2 | 163,004,157 | 163,029,875 | 1 | 25,718 | 65718 |
| [NM_013920](http://genome-www5.stanford.edu/cgi-bin/SMD/source/sourceResult?option=Number&criteria=NM_013920&choice=Gene) | Hnf4g | 3 | 3,471,064 | 3,620,337 | 1 | 149,273 | 189273 |
| [NM_008262](http://genome-www5.stanford.edu/cgi-bin/SMD/source/sourceResult?option=Number&criteria=NM_008262&choice=Gene) | HNF6 | 9 | 75,001,067 | 75,028,560 | 1 | 27,493 | 67493 |
| [NM_013556](http://genome-www5.stanford.edu/cgi-bin/SMD/source/sourceResult?option=Number&criteria=NM_013556&choice=Gene) | HPRT | X | 47,508,933 | 47,542,456 | 1 | 33,523 | 73523 |
| [NM_017371](http://genome-www5.stanford.edu/cgi-bin/SMD/source/sourceResult?option=Number&criteria=NM_017371&choice=Gene) | Hpxn | 7 | 99,704,268 | 99,713,956 | -1 | 9,688 | 49688 |
| [NM_184052](http://genome-www5.stanford.edu/cgi-bin/SMD/source/sourceResult?option=Number&criteria=NM_184052&choice=Gene) | IGF1 | 10 | 87,833,042 | 87,888,785 | 1 | 55,743 | 95743 |
| [NM_010514](http://genome-www5.stanford.edu/cgi-bin/SMD/source/sourceResult?option=Number&criteria=NM_010514&choice=Gene) | Igf2 | 7 | 137,063,788 | 137,072,509 | -1 | 8,721 |  |
| [NM_008341](http://genome-www5.stanford.edu/cgi-bin/SMD/source/sourceResult?option=Number&criteria=NM_008341&choice=Gene) | IGFBP1 | 11 | 7,092,571 | 7,097,326 | 1 | 4,755 | 44755 |
| [NM_010556](http://genome-www5.stanford.edu/cgi-bin/SMD/source/sourceResult?option=Number&criteria=NM_010556&choice=Gene) | IL-3 | 11 | 54,018,145 | 54,020,119 | -1 | 1,974 | 41974 |
| [NM_008387](http://genome-www5.stanford.edu/cgi-bin/SMD/source/sourceResult?option=Number&criteria=NM_008387&choice=Gene) | insulin2 | 7 | 137,091,680 | 137,092,728 | -1 | 1,048 | 41048 |
| [NM_008814](http://genome-www5.stanford.edu/cgi-bin/SMD/source/sourceResult?option=Number&criteria=NM_008814&choice=Gene) | ipf1 | 5 | 146,161,622 | 146,167,343 | 1 | 5,721 |  |
|  | ipf1-cdx2 | 5 |  |  | NA | 37,124 | 97124 |
| [NM_010570](http://genome-www5.stanford.edu/cgi-bin/SMD/source/sourceResult?option=Number&criteria=NM_010570&choice=Gene) | IRS1 | 1 | 82,571,724 | 82,626,531 | -1 | 54,807 | 94807 |
| [AK155277](http://genome.ucsc.edu/cgi-bin/hgc?hgsid=73383497&g=htcDisplayMrna&i=AK155277&c=chr8&l=10358729&r=10380003&o=mrna&table=mrna) | Irs2 | 8 | 10,358,730 | 10,380,003 | -1 | 21,273 | 61273 |
| [NM_021459](http://genome-www5.stanford.edu/cgi-bin/SMD/source/sourceResult?option=Number&criteria=NM_021459&choice=Gene) | Isl1 | 13 | 112,748,913 | 112,760,634 | -1 | 11,721 | 51721 |
| [NM_010637](http://genome-www5.stanford.edu/cgi-bin/SMD/source/sourceResult?option=Number&criteria=NM_010637&choice=Gene) | Klf4 | 4 | 55,443,148 | 55,448,346 | -1 | 5,198 | 45198 |
| [NM_009769](http://genome-www5.stanford.edu/cgi-bin/SMD/source/sourceResult?option=Number&criteria=NM_009769&choice=Gene) | Klf5 | 14 | 93,823,547 | 93,838,255 | 1 | 14,708 | 54708 |
| [NM_010659](http://genome-www5.stanford.edu/cgi-bin/SMD/source/sourceResult?option=Number&criteria=NM_010659&choice=Gene) | krt1-1 | 11 | 99,867,733 | 99,871,639 | -1 | 3,906 | 43906 |
| [NM_010660](http://genome-www5.stanford.edu/cgi-bin/SMD/source/sourceResult?option=Number&criteria=NM_010660&choice=Gene) | krt1-10 | 11 | 99,206,349 | 99,210,449 | -1 | 4,100 | 44100 |
| [NM_010663](http://genome-www5.stanford.edu/cgi-bin/SMD/source/sourceResult?option=Number&criteria=NM_010663&choice=Gene) | Krt1-17 | 11 | 100,077,306 | 100,082,077 | -1 | 4,771 | 44771 |
| [NM_010664](http://genome-www5.stanford.edu/cgi-bin/SMD/source/sourceResult?option=Number&criteria=NM_010664&choice=Gene) | Krt1-18 | 15 | 102,087,585 | 102,091,382 | 1 | 3,797 | 43797 |
| [NM_008471](http://genome-www5.stanford.edu/cgi-bin/SMD/source/sourceResult?option=Number&criteria=NM_008471&choice=Gene) | krt19 | 11 | 99,961,898 | 99,967,007 | -1 | 5,109 | 45109 |
| [NM_023256](http://genome-www5.stanford.edu/cgi-bin/SMD/source/sourceResult?option=Number&criteria=NM_023256&choice=Gene) | Krt20 | 11 | 99,249,490 | 99,259,241 | -1 | 9,751 | 49751 |
| [NM_008473](http://genome-www5.stanford.edu/cgi-bin/SMD/source/sourceResult?option=Number&criteria=NM_008473&choice=Gene) | krt2-1 | 15 | 101,903,891 | 101,909,249 | -1 | 5,358 | 45358 |
| [NM_008490](http://genome-www5.stanford.edu/cgi-bin/SMD/source/sourceResult?option=Number&criteria=NM_008490&choice=Gene) | LCAT | 8 | 105235400 | 105239230 | -1 | 3,830 | 43830 |
| [NM_008498](http://genome-www5.stanford.edu/cgi-bin/SMD/source/sourceResult?option=Number&criteria=NM_008498&choice=Gene) | Lhx1 | 11 | 84,247,371 | 84,253,527 | -1 | 6,156 | 46156 |
| [NM_008522](http://genome-www5.stanford.edu/cgi-bin/SMD/source/sourceResult?option=Number&criteria=NM_008522&choice=Gene) | Ltf | 9 | 111,060,590 | 111,084,064 | 1 | 23,474 | 63474 |
| [NM_017372](http://genome-www5.stanford.edu/cgi-bin/SMD/source/sourceResult?option=Number&criteria=NM_017372&choice=Gene) | Lyzs | 10 | 116,966,785 | 116,971,716 | -1 | 4,931 | 44931 |
| [NM_001025577](http://genome-www5.stanford.edu/cgi-bin/SMD/source/sourceResult?option=Number&criteria=NM_001025577&choice=Gene) | Maf | 8 | 115,018,580 | 115,021,298 | -1 | 2,718 | 42718 |
| [NM_194350](http://genome-www5.stanford.edu/cgi-bin/SMD/source/sourceResult?option=Number&criteria=NM_194350&choice=Gene) | MafA | 15 | 75796913 | 75797992 | -1 | 1,079 | 41079 |
| [NM_010658](http://genome-www5.stanford.edu/cgi-bin/SMD/source/sourceResult?option=Number&criteria=NM_010658&choice=Gene) | Mafb | 2 | 159,820,732 | 159,823,972 | -1 | 3,240 | 43240 |
| [NM_133653](http://genome-www5.stanford.edu/cgi-bin/SMD/source/sourceResult?option=Number&criteria=NM_133653&choice=Gene) | Mat1a | 14 | 39,252,260 | 39,271,290 | 1 | 19,030 | 59030 |
| [NM_007500](http://genome-www5.stanford.edu/cgi-bin/SMD/source/sourceResult?option=Number&criteria=NM_007500&choice=Gene) | Math-1 | 6 | 64,966,068 | 64,968,145 | 1 | 2,077 | 42077 |
| [NM_010800](http://genome-www5.stanford.edu/cgi-bin/SMD/source/sourceResult?option=Number&criteria=NM_010800&choice=Gene) | mist1 | 5 | 143,231,899 | 143,236,054 | 1 | 4,155 | 44155 |
| [NM_013729](http://genome-www5.stanford.edu/cgi-bin/SMD/source/sourceResult?option=Number&criteria=NM_013729&choice=Gene) | Mixl1 | 1 | 180,646,971 | 180,650,956 | -1 | 3,985 | 43985 |
| [NM_010810](http://genome-www5.stanford.edu/cgi-bin/SMD/source/sourceResult?option=Number&criteria=NM_010810&choice=Gene) | mmp7 | 9 | 7,686,777 | 7,693,931 | 1 | 7,154 | 47154 |
| [NM_008243](http://genome-www5.stanford.edu/cgi-bin/SMD/source/sourceResult?option=Number&criteria=NM_008243&choice=Gene) | Mst1 | 9 | 108,146,355 | 108,150,896 | 1 | 4,541 | 44541 |
| [NM_013605](http://genome-www5.stanford.edu/cgi-bin/SMD/source/sourceResult?option=Number&criteria=NM_013605&choice=Gene) | muc1 | 3 | 88,985,525 | 88,989,842 | 1 | 4,317 | 44317 |
| [BC051947](http://genome.ucsc.edu/cgi-bin/hgc?hgsid=73535192&g=mrna&i=BC051947&c=chr5&o=136103128&l=136103128&r=136159399&db=mm8) | Mucin-2 | 5 | 136,148,082 | 136,157,034 | -1 | 8,952 | 48952 |
| [AY455282](http://genome.ucsc.edu/cgi-bin/hgc?hgsid=73535192&g=htcDisplayMrna&i=AY455282&c=chr6&l=122677152&r=122678335&o=mrna&table=mrna) | Nanog | 6 | 123,374,566 | 123,381,605 | 1 | 7,039 | 47039 |
| [NM_010894](http://genome-www5.stanford.edu/cgi-bin/SMD/source/sourceResult?option=Number&criteria=NM_010894&choice=Gene) | NeuroD1 | 2 | 79,150,390 | 79,154,386 | -1 | 3,996 | 43996 |
| [NM_010905](http://genome-www5.stanford.edu/cgi-bin/SMD/source/sourceResult?option=Number&criteria=NM_010905&choice=Gene) | Nfia | 4 | 96,752,972 | 97,087,466 | 1 | 334,494 | 374494 |
| [NM_008687](http://genome-www5.stanford.edu/cgi-bin/SMD/source/sourceResult?option=Number&criteria=NM_008687&choice=Gene) | Nfib | 4 | 81,281,923 | 81,491,316 | -1 | 209,393 | 249393 |
| [NM_008688](http://genome-www5.stanford.edu/cgi-bin/SMD/source/sourceResult?option=Number&criteria=NM_008688&choice=Gene) | Nfic | 10 | 81,536,260 | 81,554,534 | -1 | 18,274 | 58274 |
| [NM_010906](http://genome-www5.stanford.edu/cgi-bin/SMD/source/sourceResult?option=Number&criteria=NM_010906&choice=Gene) | Nfix | 8 | 83,974,112 | 84,039,256 | -1 | 65,144 | 105144 |
| [NM_009719](http://genome-www5.stanford.edu/cgi-bin/SMD/source/sourceResult?option=Number&criteria=NM_009719&choice=Gene) | ngn3 | 10 | 62,099,692 | 62,101,366 | 1 | 1,674 | 41674 |
| [NM_010919](http://genome-www5.stanford.edu/cgi-bin/SMD/source/sourceResult?option=Number&criteria=NM_010919&choice=Gene) | Nkx2.2 | 2 | 146,640,088 | 146,643,296 | -1 | 3,208 | 43208 |
| [NM_144955](http://genome-www5.stanford.edu/cgi-bin/SMD/source/sourceResult?option=Number&criteria=NM_144955&choice=Gene) | Nkx6.1 | 5 | 100,679,979 | 100,685,483 | -1 | 5,504 | 45504 |
| [NM_008714](http://genome-www5.stanford.edu/cgi-bin/SMD/source/sourceResult?option=Number&criteria=NM_008714&choice=Gene) | Notch1 | 2 | 26,390,198 | 26,435,800 | -1 | 45,602 | 85602 |
| [NM_010928](http://genome-www5.stanford.edu/cgi-bin/SMD/source/sourceResult?option=Number&criteria=NM_010928&choice=Gene) | Notch2 | 3 | 97,499,747 | 97,633,659 | 1 | 133,912 | 173912 |
| [NM_008716](http://genome-www5.stanford.edu/cgi-bin/SMD/source/sourceResult?option=Number&criteria=NM_008716&choice=Gene) | Notch3 | 17 | 29,928,825 | 29,975,692 | -1 | 46,867 | 86867 |
| [NM_009697](http://genome-www5.stanford.edu/cgi-bin/SMD/source/sourceResult?option=Number&criteria=NM_009697&choice=Gene) | Nr2f2 | 7 | 64237866 | 64245020 | -1 | 7,154 | 47154 |
| [NM_011387](http://genome-www5.stanford.edu/cgi-bin/SMD/source/sourceResult?option=Number&criteria=NM_011387&choice=Gene) | Ntcp | 12 | 77,816,748 | 77,829,241 | -1 | 12,493 | 52493 |
| [NM_024435](http://genome-www5.stanford.edu/cgi-bin/SMD/source/sourceResult?option=Number&criteria=NM_024435&choice=Gene) | Nts | 10 | 102395460 | 102404121 | -1 | 8,661 | 48661 |
| [NM_021471](http://genome-www5.stanford.edu/cgi-bin/SMD/source/sourceResult?option=Number&criteria=NM_021471&choice=Gene) | Oatp1 | 6 | 142,330,181 | 142,375,354 | 1 | 45,173 | 85173 |
| [NM_030687](http://genome-www5.stanford.edu/cgi-bin/SMD/source/sourceResult?option=Number&criteria=NM_030687&choice=Gene) | oatp2 | 6 | 142,613,255 | 142,664,335 | -1 | 51,080 | 91080 |
| [NM_016921](http://genome-www5.stanford.edu/cgi-bin/SMD/source/sourceResult?option=Number&criteria=NM_016921&choice=Gene) | OC1 | 19 | 3,684,844 | 3,694,021 | -1 | 9,177 | 49177 |
| [NM_011144](http://genome-www5.stanford.edu/cgi-bin/SMD/source/sourceResult?option=Number&criteria=NM_011144&choice=Gene) | Oct3/4 | 17 | 33,222,929 | 33,228,085 | 1 | 5,156 | 45156 |
| [NM_194268](http://genome-www5.stanford.edu/cgi-bin/SMD/source/sourceResult?option=Number&criteria=NM_194268&choice=Gene) | Onecut2 | 18 | 64,571,908 | 64,618,373 | 1 | 46,465 | 86465 |
| [NM_008768](http://genome-www5.stanford.edu/cgi-bin/SMD/source/sourceResult?option=Number&criteria=NM_008768&choice=Gene) | orm1 | 4 | 62,435,758 | 62,439,354 | 1 | 3,596 | 43596 |
| [NM_144841](http://genome-www5.stanford.edu/cgi-bin/SMD/source/sourceResult?option=Number&criteria=NM_144841&choice=Gene) | Otx2 | 14 | 43,746,617 | 43,754,045 | -1 | 7,428 | 47428 |
| [NM_008777](http://genome-www5.stanford.edu/cgi-bin/SMD/source/sourceResult?option=Number&criteria=NM_008777&choice=Gene) | PAH | 10 | 87,495,518 | 87,557,285 | 1 | 61,767 | 101767 |
| [NM_011038](http://genome-www5.stanford.edu/cgi-bin/SMD/source/sourceResult?option=Number&criteria=NM_011038&choice=Gene) | Pax4 | 6 | 28,489,335 | 28,496,271 | -1 | 6,936 | 46936 |
| [NM_013627](http://genome-www5.stanford.edu/cgi-bin/SMD/source/sourceResult?option=Number&criteria=NM_013627&choice=Gene) | Pax6 | 2 | 105373665 | 105402118 | 1 | 28,453 | 68453 |
| [NM_008783](http://genome-www5.stanford.edu/cgi-bin/SMD/source/sourceResult?option=Number&criteria=NM_008783&choice=Gene) | Pbx1 | 1 | 168,054,563 | 168,366,387 | -1 | 311,824 | 351824 |
| [NM_011058](http://genome-www5.stanford.edu/cgi-bin/SMD/source/sourceResult?option=Number&criteria=NM_011058&choice=Gene) | pdgfra | 5 | 73,986,573 | 74,032,425 | 1 | 45,852 | 85852 |
| [NM_011044](http://genome-www5.stanford.edu/cgi-bin/SMD/source/sourceResult?option=Number&criteria=NM_011044&choice=Gene) | PEPCK | 2 | 172,613,540 | 172,619,719 | 1 | 6,179 | 46179 |
| [NM_011097](http://genome-www5.stanford.edu/cgi-bin/SMD/source/sourceResult?option=Number&criteria=NM_011097&choice=Gene) | Pitx | 13 | 54,441,613 | 54,447,984 | -1 | 6,371 | 46371 |
| [NM_011144](http://genome-www5.stanford.edu/cgi-bin/SMD/source/sourceResult?option=Number&criteria=NM_011144&choice=Gene) | PPARa | 15 | 85,784,420 | 85,851,463 | 1 | 67,043 | 107043 |
| [NM_008918](http://genome-www5.stanford.edu/cgi-bin/SMD/source/sourceResult?option=Number&criteria=NM_008918&choice=Gene) | Ppy | 11 | 101,921,018 | 101,922,388 | -1 | 1,370 | 41370 |
| [NM_008934](http://genome-www5.stanford.edu/cgi-bin/SMD/source/sourceResult?option=Number&criteria=NM_008934&choice=Gene) | Proc | 18 | 32,363,271 | 32,376,084 | -1 | 12,813 | 52813 |
| [NM_008937](http://genome-www5.stanford.edu/cgi-bin/SMD/source/sourceResult?option=Number&criteria=NM_008937&choice=Gene) | Prox1 | 1 | 189,836,620 | 189,885,752 | -1 | 49,132 | 89132 |
| [NM_008957](http://genome-www5.stanford.edu/cgi-bin/SMD/source/sourceResult?option=Number&criteria=NM_008957&choice=Gene) | Ptch1 | 13 | 60,894,523 | 60,948,511 | -1 | 53,988 | 93988 |
| [NM_018809](http://genome-www5.stanford.edu/cgi-bin/SMD/source/sourceResult?option=Number&criteria=NM_018809&choice=Gene) | Ptf1a | 2 | 19,487,673 | 19,489,505 | 1 | 1,832 | 41832 |
| [NM_145435](http://genome-www5.stanford.edu/cgi-bin/SMD/source/sourceResult?option=Number&criteria=NM_145435&choice=Gene) | Pyy | 11 | 101,927,763 | 101,928,864 | -1 | 1,101 | 41101 |
| [NM_009117](http://genome-www5.stanford.edu/cgi-bin/SMD/source/sourceResult?option=Number&criteria=NM_009117&choice=Gene) | SAA1 | 7 | 40,825,849 | 40,828,329 | -1 | 2,480 | 42480 |
| [NM_011314](http://genome-www5.stanford.edu/cgi-bin/SMD/source/sourceResult?option=Number&criteria=NM_011314&choice=Gene) | saa2 | 7 | 40,837,181 | 40,839,661 | 1 | 2,480 | 42480 |
| [NM_011315](http://genome-www5.stanford.edu/cgi-bin/SMD/source/sourceResult?option=Number&criteria=NM_011315&choice=Gene) | Saa3 | 7 | 40,797,347 | 40,801,025 | -1 | 3,678 | 43678 |
| [NM_011316](http://genome-www5.stanford.edu/cgi-bin/SMD/source/sourceResult?option=Number&criteria=NM_011316&choice=Gene) | saa4 | 7 | 40,813,347 | 40,817,893 | -1 | 4,546 | 44546 |
| [NM_172205](http://genome-www5.stanford.edu/cgi-bin/SMD/source/sourceResult?option=Number&criteria=NM_172205&choice=Gene) | Sbsn | 7 | 26,160,849 | 26,165,449 | 1 | 4,600 | 44600 |
| [NM_011328](http://genome-www5.stanford.edu/cgi-bin/SMD/source/sourceResult?option=Number&criteria=NM_011328&choice=Gene) | Sct | 7 | 135,680,317 | 135,681,094 | -1 | 777 | 40777 |
| [NM_009246](http://genome-www5.stanford.edu/cgi-bin/SMD/source/sourceResult?option=Number&criteria=NM_009246&choice=Gene) | Serpina1d | 12 | 99,207,541 | 99,217,517 | -1 | 9,976 | 49976 |
| [NM_023134](http://genome-www5.stanford.edu/cgi-bin/SMD/source/sourceResult?option=Number&criteria=NM_023134&choice=Gene) | Sftpa1 | 14 | 39,278,667 | 39,283,253 | 1 | 4,586 | 44586 |
| [NM_147779](http://genome-www5.stanford.edu/cgi-bin/SMD/source/sourceResult?option=Number&criteria=NM_147779&choice=Gene) | Sftpb | 6 | 72,635,222 | 72,646,345 | 1 | 11,123 | 51123 |
| [NM_011359](http://genome-www5.stanford.edu/cgi-bin/SMD/source/sourceResult?option=Number&criteria=NM_011359&choice=Gene) | Sftpc | 14 | 64,836,929 | 64,840,060 | -1 | 3,131 | 43131 |
| [NM_009170](http://genome-www5.stanford.edu/cgi-bin/SMD/source/sourceResult?option=Number&criteria=NM_009170&choice=Gene) | Shh | 5 | 26,906,865 | 26,917,113 | -1 | 10,248 | 50248 |
| [NM_009203](http://genome-www5.stanford.edu/cgi-bin/SMD/source/sourceResult?option=Number&criteria=NM_009203&choice=Gene) | Slc22a12 | 19 | 6,325,217 | 6,332,366 | -1 | 7,149 | 47149 |
| [NM_031197](http://genome-www5.stanford.edu/cgi-bin/SMD/source/sourceResult?option=Number&criteria=NM_031197&choice=Gene) | Slc2a2 | 3 | 28115361 | 28145712 | 1 | 30,351 | 70351 |
| [NM_010754](http://genome-www5.stanford.edu/cgi-bin/SMD/source/sourceResult?option=Number&criteria=NM_010754&choice=Gene) | Smad2 | 18 | 76,478,323 | 76,539,931 | 1 | 61,608 | 101608 |
| [NM_008540](http://genome-www5.stanford.edu/cgi-bin/SMD/source/sourceResult?option=Number&criteria=NM_008540&choice=Gene) | Smad4 | 18 | 73,873,243 | 73,937,971 | -1 | 64,728 | 104728 |
| [NM_009233](http://genome-www5.stanford.edu/cgi-bin/SMD/source/sourceResult?option=Number&criteria=NM_009233&choice=Gene) | Sox1 | 8 | 11,774,868 | 11,776,044 | 1 | 1,176 | 41176 |
| [NM_011441](http://genome-www5.stanford.edu/cgi-bin/SMD/source/sourceResult?option=Number&criteria=NM_011441&choice=Gene) | sox17 | 1 | 4,451,317 | 4,456,803 | -1 | 5,486 | 45486 |
| [NM_011443](http://genome-www5.stanford.edu/cgi-bin/SMD/source/sourceResult?option=Number&criteria=NM_011443&choice=Gene) | Sox2 | 3 | 34105755 | 34108159 | 1 | 2,404 | 42404 |
| [NM_011480](http://genome-www5.stanford.edu/cgi-bin/SMD/source/sourceResult?option=Number&criteria=NM_011480&choice=Gene) | SREBP-1c | 11 | 59,924,731 | 59,946,247 | -1 | 21,516 | 61516 |
| [NM_009215](http://genome-www5.stanford.edu/cgi-bin/SMD/source/sourceResult?option=Number&criteria=NM_009215&choice=Gene) | Sst | 16 | 22,673,099 | 22,674,363 | -1 | 1,264 | 41264 |
| [NM_146214](http://genome-www5.stanford.edu/cgi-bin/SMD/source/sourceResult?option=Number&criteria=NM_146214&choice=Gene) | TAT | 8 | 109,288,133 | 109,297,477 | 1 | 9,344 | 49344 |
| [NM_011575](http://genome-www5.stanford.edu/cgi-bin/SMD/source/sourceResult?option=Number&criteria=NM_011575&choice=Gene) | Tff3 | 17 | 28,939,315 | 28,943,612 | -1 | 4,297 | 44297 |
| [NM_011638](http://genome-www5.stanford.edu/cgi-bin/SMD/source/sourceResult?option=Number&criteria=NM_011638&choice=Gene) | Tfrc | 16 | 31,419,658 | 31,443,429 | 1 | 23,771 | 63771 |
| [NM_009382](http://genome-www5.stanford.edu/cgi-bin/SMD/source/sourceResult?option=Number&criteria=NM_009382&choice=Gene) | thy1 | 9 | 44,034,810 | 44,037,435 | 1 | 2,625 | 42625 |
| [NM_009385](http://genome-www5.stanford.edu/cgi-bin/SMD/source/sourceResult?option=Number&criteria=NM_009385&choice=Gene) | Titf1 | 12 | 53257523 | 53262475 | -1 | 4,952 | 44952 |
| [NM_011599](http://genome-www5.stanford.edu/cgi-bin/SMD/source/sourceResult?option=Number&criteria=NM_011599&choice=Gene) | TLE1 | 4 | 71,248,775 | 71,292,056 | -1 | 43,281 | 83281 |
| [NM_019725](http://genome-www5.stanford.edu/cgi-bin/SMD/source/sourceResult?option=Number&criteria=NM_019725&choice=Gene) | TLE2 | 10 | 81,711,856 | 81,727,531 | 1 | 15,675 | 55675 |
| [NM_009389](http://genome-www5.stanford.edu/cgi-bin/SMD/source/sourceResult?option=Number&criteria=NM_009389&choice=Gene) | TLE3 | 9 | 61,529,817 | 61,532,261 | 1 | 2,444 | 42444 |
| [NM_011600](http://genome-www5.stanford.edu/cgi-bin/SMD/source/sourceResult?option=Number&criteria=NM_011600&choice=Gene) | TLE4 | 19 | 13,671,934 | 13,821,846 | -1 | 149,912 | 189912 |
| [NM_025382](http://genome-www5.stanford.edu/cgi-bin/SMD/source/sourceResult?option=Number&criteria=NM_025382&choice=Gene) | Tmem57 | 4 | 133,763,666 | 133,814,034 | -1 | 50,368 | 90368 |
| [NM_133977](http://genome-www5.stanford.edu/cgi-bin/SMD/source/sourceResult?option=Number&criteria=NM_133977&choice=Gene) | Trf | 9 | 103184318 | 103205728 | -1 | 21,410 | 61410 |
| [NM_013697](http://genome-www5.stanford.edu/cgi-bin/SMD/source/sourceResult?option=Number&criteria=NM_013697&choice=Gene) | Ttr | 18 | 20,880,341 | 20,889,246 | 1 | 8,905 | 48905 |
| [NM_011653](http://genome-www5.stanford.edu/cgi-bin/SMD/source/sourceResult?option=Number&criteria=NM_011653&choice=Gene) | tuba1 | 15 | 99,007,626 | 99,011,283 | -1 | 3,657 | 43657 |
| [NM_009450](http://genome-www5.stanford.edu/cgi-bin/SMD/source/sourceResult?option=Number&criteria=NM_009450&choice=Gene) | tubb2 | 13 | 33,608,791 | 33,612,496 | -1 | 3,705 | 43705 |
| [NM_009470](http://genome-www5.stanford.edu/cgi-bin/SMD/source/sourceResult?option=Number&criteria=NM_009470&choice=Gene) | Umod | 7 | 113,338,426 | 113,354,816 | -1 | 16,390 | 56390 |
| [NM_009505](http://genome-www5.stanford.edu/cgi-bin/SMD/source/sourceResult?option=Number&criteria=NM_009505&choice=Gene) | Vegfa | 17 | 43,526,917 | 43,541,672 | -1 | 14,755 | 54755 |
| [NM_144783](http://genome-www5.stanford.edu/cgi-bin/SMD/source/sourceResult?option=Number&criteria=NM_144783&choice=Gene) | Wt1 | 2 | 104,831,283 | 104,878,367 | 1 | 47,084 | 87084 |
| [NM_013842](http://genome-www5.stanford.edu/cgi-bin/SMD/source/sourceResult?option=Number&criteria=NM_013842&choice=Gene) | xbp-1 | 11 | 5,415,429 | 5,420,658 | 1 | 5,229 | 45229 |
