# Supplementary material for: Study of FoxA Pioneer Factor at Silent Genes Reveals Rfx-Repressed Enhancer at Cdx2 and a Potential Indicator of Esophageal Adenocarcinoma Development
Source: PLoS Genet. 2011 Sep 15;7(9):e1002277. doi: 10.1371/journal.pgen.1002277 (PMC3174211; doi:10.1371/journal.pgen.1002277)
Supplement: Table S2 — Liver FoxA Binding Sites. (DOCX) [file pgen.1002277.s007.docx]

| **Supplemental Table 2: Liver FoxA Binding Sites** | | |  |  |  |  |  |
| --- | --- | --- | --- | --- | --- | --- | --- |
|  |  |  |  |  |  |  |  |
| **Gene Name** | **FoxA Bound Region** | **MAT** | **Tss** | **distanceToTss** | **location** | **matchStrand** | **conservation** |
| Fga | chr3:82749111-82750089 | 29.3 | 82755131 | 5531 | 5'end | + | 0.3626375 |
| TLE1 | chr4:71289446-71290623 | 28.7 | 71292055 | 2021 | inside | - | 0.32726 |
| Sftpb | chr6:72624693-72625654 | 25.2 | 72635222 | 10049 | 5'end | + | 0.3163425 |
| AFM | chr5:89858104-89858923 | 24.1 | 89816391 | 42122 | 3'end | + | 0.2016475 |
| Prox1 | chr1:189836861-189837750 | 23.4 | 189885751 | 48446 | inside | - | 0.2103625 |
| PPARa | chr15:85819841-85821249 | 22.6 | 85784420 | 36125 | inside | + | 0.066665 |
| Gc | chr5:88765650-88766203 | 22.2 | 88756681 | 9245 | 5'end | - | 0.20232 |
| Ntcp | chr12:77837141-77837814 | 21.5 | 77829240 | 8237 | 5'end | - | 0.185095 |
| Arg1 | chr10:24925796-24926565 | 21.3 | 24903676 | 22504 | 5'end | - | 0.135345 |
| Gna1 | chr18:67446004-67446797 | 20.5 | 67317670 | 128730 | inside | + | 0.3886375 |
| glut2 | chr3:28099043-28099860 | 20.0 | 28115361 | 15910 | 5'end | + | 0.491155 |
| PEPCK | chr2:172584662-172585383 | 19.8 | 172613540 | 28518 | 5'end | + | 0.1652775 |
| Saa3 | chr7:40804786-40805483 | 19.8 | 40801024 | 4110 | 5'end | - | 0.10504 |
| apob | chr12:7195355-7196172 | 19.7 | 7223049 | 27286 | 5'end | + | 0.4218875 |
| Notch2 | chr3:97531009-97531517 | 19.6 | 97499747 | 31516 | inside | + | 0.544595 |
| apob | chr12:7223583-7224424 | 19.5 | 7223049 | 954 | inside | + | 0.2739875 |
| IGFBP1 | chr11:7089079-7089800 | 18.9 | 7092571 | 3132 | 5'end | + | 0.1396275 |
| Prox1 | chr1:189844230-189844975 | 18.9 | 189885751 | 41149 | inside | - | 0.553295 |
| TLE3 | chr9:61508781-61509430 | 18.7 | 61529817 | 20712 | 5'end | + | 0.3428875 |
| Ttr | chr18:20879853-20880550 | 18.7 | 20880341 | 140 | 5'end | + | 0.3918 |
| Oatp1 | chr6:142328277-142328638 | 18.5 | 142330181 | 1724 | 5'end | + | 0.1304175 |
| Trf | chr9:103209764-103210505 | 18.5 | 103205727 | 4407 | 5'end | - | 0.237895 |
| apob | chr12:7222596-7223272 | 18.3 | 7223049 | 115 | 5'end | + | 0.274315 |
| Onecut2 | chr18:64557002-64557683 | 18.2 | 64571908 | 14566 | 5'end | + | 0.092085 |
| OC1 | chr19:3701646-3702439 | 18.1 | 3694020 | 8022 | 5'end | - | 0.28645 |
| IGF1 | chr10:87833900-87834645 | 17.7 | 87833042 | 1230 | inside | + | 0.62033 |
| SREBP-1c | chr11:59966064-59966617 | 17.7 | 59946246 | 20094 | 5'end | - | 0.293915 |
| Mat1a | chr14:39247590-39248537 | 17.7 | 39252260 | 4197 | 5'end | + | 0.07972 |
| Serpina1d | chr12:99217292-99218085 | 17.7 | 99217516 | 172 | 5'end | - | 0.269585 |
| IGF1 | chr10:87888579-87889063 | 17.2 | 87833042 | 55779 | 3'end | + | 0.18441 |
| FoxA2 | chr2:147497008-147497658 | 17.0 | 147503905 | 6572 | 3'end | - | 0.79891 |
| Mat1a | chr14:39242991-39243568 | 17.0 | 39252260 | 8981 | 5'end | + | 0.18860294 |
| Gata4 | chr14:57735805-57736454 | 16.9 | 57775851 | 39722 | inside | - | 0.1057325 |
| Dpp4 | chr2:62238328-62238929 | 16.9 | 62267482 | 28854 | inside | - | 0.1369375 |
| Hex | chr19:36785682-36786379 | 16.9 | 36778120 | 7910 | 3'end | + | 0.891115 |
| Nfib | chr4:81382925-81383609 | 16.6 | 81491315 | 108048 | inside | - | 0.2584875 |
| HEs1 | chr16:28858249-28858880 | 16.5 | 28870268 | 11704 | 5'end | + | 0.4005675 |
| cdx2 | chr5:146190864-146191609 | 16.5 | 146198744 | 7508 | 3'end | - | 0.46589 |
| Fga | chr3:82731975-82732669 | 16.4 | 82755131 | 22809 | 5'end | + | 0.2655725 |
| Apoa4 | chr9:46269658-46270456 | 16.3 | 46254406 | 15651 | 3'end | + | 0.1902025 |
| FoxA2 | chr2:147503616-147504405 | 16.3 | 147503905 | 105 | 5'end | - | 0.3763925 |
| Maf | chr8:115033229-115033855 | 16.3 | 115021297 | 12245 | 5'end | - | 0.113875 |
| TLE1 | chr4:71256732-71257189 | 16.2 | 71292055 | 35095 | inside | - | 0.18969388 |
| xbp-1 | chr11:5393722-5394467 | 16.0 | 5415429 | 21335 | 5'end | + | 0.20271 |
| ApoA1 | chr9:46228735-46229432 | 15.9 | 46241962 | 12879 | 5'end | + | 0.27619 |
| Apoa2 | chr1:171153563-171154164 | 15.9 | 171153928 | 65 | 5'end | + | 0.1224025 |
| Notch2 | chr3:97503337-97503722 | 15.8 | 97499747 | 3782 | inside | + | 0.1524475 |
| Cdh16 | chr8:103933465-103934114 | 15.8 | 103917396 | 16393 | 5'end | - | 0.18882 |
| Fabp1 | chr6:71504448-71505102 | 15.7 | 71531814 | 27039 | 5'end | + | 0.12235 |
| Hpxn | chr7:99713746-99714419 | 15.7 | 99713955 | 127 | 5'end | - | 0.34468 |
| Vegfa | chr17:43561109-43561806 | 15.6 | 43541671 | 19786 | 5'end | - | 0.5649525 |
| Nfix | chr8:84037419-84038195 | 15.6 | 84039255 | 1448 | inside | - | 0.6351025 |
| Nfic | chr10:81555867-81556420 | 15.5 | 81554533 | 1610 | 5'end | - | 0.2397025 |
| TAT | chr8:109281736-109282301 | 15.5 | 109288133 | 6115 | 5'end | + | 0.1979075 |
| Nfia | chr4:96886093-96886791 | 15.5 | 96752972 | 133470 | inside | + | 0.3033675 |
| Notch2 | chr3:97512018-97512737 | 15.4 | 97499747 | 12630 | inside | + | 0.153305 |
| Tff3 | chr17:28952433-28952986 | 15.4 | 28943611 | 9098 | 5'end | - | 0.1631225 |
| Ttr | chr18:20856059-20856804 | 15.4 | 20880341 | 23910 | 5'end | + | 0.1273075 |
| gastrin | chr11:100136603-100137060 | 15.3 | 100155494 | 18663 | 5'end | + | 0.2047825 |
| PAH | chr10:87493824-87494377 | 15.2 | 87495518 | 1418 | 5'end | + | 0.11578 |
| Krt20 | chr11:99269778-99270403 | 15.1 | 99259240 | 10850 | 5'end | - | 0.1735725 |
| AldoB | chr4:49454435-49455036 | 15.0 | 49465357 | 10622 | inside | - | 0.4757175 |
| Fabp2 | chr3:121679154-121679755 | 15.0 | 121686438 | 6984 | 5'end | + | 0.1935225 |
| AldoB | chr4:49473920-49474329 | 14.9 | 49465357 | 8767 | 5'end | - | 0.2551225 |
| Igf2 | chr7:137035071-137035696 | 14.9 | 137072508 | 37125 | 3'end | - | 0.369225 |
| xbp-1 | chr11:5386620-5387077 | 14.8 | 5415429 | 28581 | 5'end | + | 0.2795075 |
| Fgfr2 | chr7:124345601-124346130 | 14.8 | 124389452 | 43587 | inside | - | 0.295005 |
| Mixl1 | chr1:180668852-180669308 | 14.8 | 180650955 | 18125 | 5'end | - | 0.1816901 |
| Gata4 | chr14:57761844-57762445 | 14.7 | 57775851 | 13707 | inside | - | 0.6433275 |
| Fabp1 | chr6:71516929-71517554 | 14.6 | 71531814 | 14573 | 5'end | + | 0.23205 |
| TAT | chr8:109290596-109291005 | 14.6 | 109288133 | 2667 | inside | + | 0.168525 |
| CAR | chr1:171132992-171133568 | 14.6 | 171142841 | 9561 | 5'end | + | 0.2075225 |
| TAT | chr8:109276929-109277338 | 14.4 | 109288133 | 11000 | 5'end | + | 0.2185275 |
| chrd | chr16:19504579-19505168 | 14.4 | 19504234 | 639 | inside | + | 0.464645 |
| Hnf4a | chr2:162997427-162998028 | 14.4 | 163004157 | 6430 | 5'end | + | 0.4917225 |
| Arg1 | chr10:24899142-24899791 | 14.4 | 24903676 | 4210 | inside | - | 0.103335 |
| cKit | chr5:74430500-74431065 | 14.3 | 74409245 | 21537 | inside | + | 0.08284524 |
| CRP | chr1:172626466-172626899 | 14.3 | 172626985 | 303 | 5'end | + | 0.20453 |
| PPARa | chr15:85781261-85781910 | 14.2 | 85784420 | 2835 | 5'end | + | 0.3748475 |
| SREBP-1c | chr11:59972828-59973117 | 14.2 | 59946246 | 26726 | 5'end | - | 0.069295 |
| AFM | chr5:89866246-89866847 | 14.1 | 89816391 | 50155 | 3'end | + | 0.183065 |
| TAT | chr8:109267947-109268404 | 14.1 | 109288133 | 19958 | 5'end | + | 0.2724175 |
| G6PC | chr11:101183192-101183789 | 14.1 | 101188817 | 5327 | 5'end | + | 0.44851 |
| krt1-1 | chr11:99866800-99867353 | 14.0 | 99871638 | 4562 | 3'end | - | 0.102265 |
| HNF1beta | chr11:83609488-83609969 | 13.9 | 83579041 | 30687 | inside | + | 0.436965 |
| Arg1 | chr10:24903463-24904016 | 13.9 | 24903676 | 63 | 5'end | - | 0.250005 |
| Igf2 | chr7:137031854-137032551 | 13.8 | 137072508 | 40306 | 3'end | - | 0.07791 |
| Gli3 | chr13:14961814-14962343 | 13.8 | 14911831 | 50247 | inside | + | 0.0994525 |
| Fgfr2 | chr7:124371644-124372173 | 13.8 | 124389452 | 17544 | inside | - | 0.0592225 |
| Glepp1 | chr6:138264016-138264473 | 13.8 | 138044285 | 219959 | 3'end | + | 0.119505 |
| PPARa | chr15:85780464-85781017 | 13.8 | 85784420 | 3680 | 5'end | + | 0.26073 |
| Igf2 | chr7:137032886-137033463 | 13.7 | 137072508 | 39334 | 3'end | - | 0.07248 |
| LCAT | chr8:105237905-105238627 | 13.7 | 105239229 | 963 | inside | - | 0.4808625 |
| Nfia | chr4:96843336-96843889 | 13.7 | 96752972 | 90640 | inside | + | 0.6602 |
| AFP | chr5:89782961-89783342 | 13.7 | 89788165 | 5014 | 5'end | + | 0.16096 |
| Nfia | chr4:96979024-96979673 | 13.7 | 96752972 | 226376 | inside | + | 0.7610275 |
| cps1 | chr1:67509645-67510222 | 13.6 | 67466664 | 43269 | inside | + | 0.665025 |
| Ttr | chr18:20878217-20878746 | 13.6 | 20880341 | 1860 | 5'end | + | 0.12842 |
| Gata5 | chr2:180060716-180061245 | 13.5 | 180051631 | 9349 | 5'end | - | 0.12245 |
| HNF1beta | chr11:83641799-83642472 | 13.5 | 83579041 | 63094 | 3'end | + | 0.87656 |
| Alb | chr5:89764618-89765134 | 13.5 | 89758329 | 6547 | inside | + | 0.392235 |
| Alb | chr5:89728570-89728835 | 13.5 | 89758329 | 29627 | 5'end | + | 0.54789 |
| H19 | chr7:137021777-137022292 | 13.4 | 136990034 | 32000 | 5'end | - | 0.1753575 |
| tubb2 | chr13:33611423-33611976 | 13.4 | 33612495 | 796 | inside | - | 0.3780825 |
| IGF1 | chr10:87830583-87831088 | 13.4 | 87833042 | 2207 | 5'end | + | 0.123905 |
| C/EBPa | chr7:30284952-30285213 | 13.4 | 30276207 | 8876 | 3'end | + | 0.21938 |
| oatp2 | chr6:142676894-142677231 | 13.4 | 142664334 | 12728 | 5'end | - | 0.16076267 |
| Sftpc | chr14:64831606-64832207 | 13.4 | 64840059 | 8153 | 3'end | - | 0.2641075 |
| krt1-10 | chr11:99234390-99234847 | 13.3 | 99210448 | 24170 | 5'end | - | 0.1435225 |
| Nfic | chr10:81553323-81553924 | 13.3 | 81554533 | 910 | inside | - | 0.062985 |
| Fga | chr3:82738830-82739677 | 13.3 | 82755131 | 15878 | 5'end | + | 0.25818 |
| Fgfr2 | chr7:124248006-124248607 | 13.3 | 124389452 | 141146 | inside | - | 0.1664075 |
| Lhx1 | chr11:84240629-84241134 | 13.3 | 84253526 | 12645 | 3'end | - | 0.1800675 |
| Trf | chr9:103182902-103183455 | 13.3 | 103205727 | 22549 | 3'end | - | 0.22074 |
| Trf | chr9:103219098-103219627 | 13.3 | 103205727 | 13635 | 5'end | - | 0.2236625 |
| xbp-1 | chr11:5404506-5404963 | 13.2 | 5415429 | 10695 | 5'end | + | 0.110005 |
| Fgfr2 | chr7:124369961-124370490 | 13.2 | 124389452 | 19227 | inside | - | 0.1262825 |
| glut2 | chr3:28142541-28143168 | 13.2 | 28115361 | 27493 | inside | + | 0.1647 |
| Nfia | chr4:96759282-96759854 | 13.2 | 96752972 | 6596 | inside | + | 0.4634725 |
| IRS1 | chr1:82641501-82641959 | 13.2 | 82626530 | 15200 | 5'end | - | 0.105545 |
| cdh1 | chr8:105906700-105907311 | 13.2 | 105899006 | 7999 | inside | + | 0.2172425 |
| CC10 | chr19:8287127-8287690 | 13.2 | 8284460 | 2948 | 5'end | - | 0.3172025 |
| ipf1 | chr5:146174642-146175075 | 13.1 | 146161621 | 13237 | 3'end | + | NA |
| Sst | chr16:22679127-22679512 | 13.1 | 22674362 | 4957 | 5'end | - | 0.09171 |
| HNF1beta | chr11:83605789-83606198 | 13.1 | 83579041 | 26952 | inside | + | 0.53228 |
| PPARa | chr15:85791178-85791683 | 13.0 | 85784420 | 7010 | inside | + | 0.0651575 |
| PEPCK | chr2:172613266-172613723 | 12.9 | 172613540 | 46 | 5'end | + | 0.239195 |
| FoxA1 | chr12:54275093-54275550 | 12.9 | 54277439 | 2118 | inside | - | 0.2713175 |
| Nfix | chr8:84027332-84027837 | 12.8 | 84039255 | 11671 | inside | - | 0.1681025 |
| Gata6 | chr18:11095809-11096410 | 12.8 | 11097852 | 1743 | 5'end | + | 0.3370425 |
| Vegfa | chr17:43566227-43566780 | 12.7 | 43541671 | 24832 | 5'end | - | 0.319195 |
| TLE3 | chr9:61503791-61504272 | 12.7 | 61529817 | 25786 | 5'end | + | 0.1475175 |
| ApoE | chr7:16564302-16564855 | 12.7 | 16568276 | 3698 | 3'end | - | 0.1053425 |
| Irs2 | chr8:10393468-10393949 | 12.7 | 10380002 | 13706 | 5'end | - | 0.083295 |
| HNF1beta | chr11:83625144-83625793 | 12.6 | 83579041 | 46427 | inside | + | 0.26891 |
| Isl1 | chr13:112746611-112747068 | 12.6 | 112760633 | 13794 | 3'end | - | 0.5047525 |
| Fabp2 | chr3:121686329-121686810 | 12.6 | 121686438 | 131 | inside | + | 0.3301725 |
| Onecut2 | chr18:64575201-64575611 | 12.5 | 64571908 | 3498 | inside | + | 0.33209 |
| Sbsn | chr7:26138798-26139183 | 12.5 | 26160849 | 21859 | 5'end | + | 0.27351 |
| Serpina1d | chr12:99213640-99213929 | 12.5 | 99217516 | 3732 | inside | - | 0.12402 |
| cps1 | chr1:67438323-67438787 | 12.5 | 67466664 | 28109 | 5'end | + | 0.138875 |
| apob | chr12:7229481-7229986 | 12.5 | 7223049 | 6684 | inside | + | 0.0867075 |
| Nfia | chr4:96784441-96784970 | 12.5 | 96752972 | 31733 | inside | + | 0.099405 |
| Sox2 | chr3:34114092-34114645 | 12.3 | 34105755 | 8613 | 3'end | + | 0.1529225 |
| Cp | chr3:19298131-19298509 | 12.3 | 19298453 | 133 | 5'end | + | 0.3365925 |
| AldoB | chr4:49475503-49475912 | 12.3 | 49465357 | 10350 | 5'end | - | 0.08314 |
| Umod | chr7:113366071-113366552 | 12.3 | 113354815 | 11496 | 5'end | - | 0.097125 |
| Cldn6 | chr17:21479122-21479651 | 12.2 | 21483245 | 3859 | 5'end | + | 0.21574 |
| Alb | chr5:89744912-89745337 | 12.2 | 89758329 | 13205 | 5'end | + | 0.185695 |
| TLE4 | chr19:13834640-13835219 | 12.2 | 13821845 | 13084 | 5'end | - | 0.974615 |
| HNF6 | chr9:75005707-75006260 | 12.2 | 75001067 | 4916 | inside | + | 0.3913725 |
| PPARa | chr15:85823062-85823471 | 12.2 | 85784420 | 38846 | inside | + | 0.1637375 |
| Sftpc | chr14:64856890-64857299 | 12.1 | 64840059 | 17035 | 5'end | - | 0.3491175 |
| Proc | chr18:32378306-32378690 | 12.1 | 32376083 | 2415 | 5'end | - | 0.336905 |
| krt19 | chr11:99955854-99956238 | 12.1 | 99967006 | 10960 | 3'end | - | 0.0909375 |
| Acvr2a | chr2:48748243-48748844 | 12.1 | 48746319 | 2224 | inside | + | 0.751425 |
| IGF1 | chr10:87858270-87858703 | 12.0 | 87833042 | 25444 | inside | + | 0.332405 |
| PAH | chr10:87519945-87520329 | 12.0 | 87495518 | 24619 | inside | + | 0.120475 |
| ApoE | chr7:16570063-16570447 | 12.0 | 16568276 | 1979 | 5'end | - | 0.06155 |
| CC10 | chr19:8282341-8282846 | 11.9 | 8284460 | 1867 | inside | - | 0.083545 |
| Sst | chr16:22684414-22684943 | 11.9 | 22674362 | 10316 | 5'end | - | 0.1156775 |
| Gli3 | chr13:15032366-15032775 | 11.9 | 14911831 | 120739 | inside | + | 0.06485 |
| IGF1 | chr10:87879675-87880233 | 11.9 | 87833042 | 46912 | inside | + | 0.5140725 |
| PEPCK | chr2:172608457-172608818 | 11.8 | 172613540 | 4903 | 5'end | + | 0.10258943 |
| Prox1 | chr1:189879164-189879765 | 11.8 | 189885751 | 6287 | inside | - | 0.1385975 |
| Cdh16 | chr8:103900677-103901158 | 11.8 | 103917396 | 16479 | 3'end | - | 0.28688 |
| Nr2f2 | chr7:64250301-64250782 | 11.7 | 64245019 | 5522 | 5'end | - | 0.874975 |
| Smad4 | chr18:73899665-73900098 | 11.6 | 73937970 | 38089 | inside | - | 0.04674857 |
| Notch2 | chr3:97614759-97615240 | 11.6 | 97499747 | 115252 | inside | + | 0.11251 |
| IGFBP1 | chr11:7087285-7087718 | 11.6 | 7092571 | 5070 | 5'end | + | 0.261335 |
| Nfia | chr4:97053609-97054042 | 11.6 | 96752972 | 300853 | inside | + | 0.168475 |
| cKit | chr5:74461004-74461413 | 11.6 | 74409245 | 51963 | inside | + | 0.23667 |
| Fabp2 | chr3:121663076-121663605 | 11.5 | 121686438 | 23098 | 5'end | + | 0.2831023 |
| Notch2 | chr3:97594828-97595165 | 11.4 | 97499747 | 95249 | inside | + | 0.2140925 |
| ghrelin | chr6:114278581-114278965 | 11.4 | 114288467 | 9694 | 3'end | - | 0.417875 |
| Fabp2 | chr3:121672350-121672759 | 11.3 | 121686438 | 13884 | 5'end | + | 0.188995 |
| Cyp7a1 | chr4:6199439-6199848 | 11.3 | 6202777 | 3134 | inside | - | 0.7843725 |
| Nfia | chr4:96899527-96899940 | 11.3 | 96752972 | 146761 | inside | + | 0.6768175 |
| Fgl1 | chr8:40153698-40154082 | 11.3 | 40152758 | 1132 | 5'end | - | 0.28845 |
| Apoa4 | chr9:46270839-46271272 | 11.3 | 46254406 | 16649 | 3'end | + | 0.1790525 |
| G6PC | chr11:101191464-101191855 | 11.2 | 101188817 | 2843 | inside | + | 0.2503375 |
| Nfia | chr4:96961047-96961445 | 11.2 | 96752972 | 208274 | inside | + | 0.240265 |
| Gata6 | chr18:11124758-11125095 | 11.2 | 11097852 | 27074 | inside | + | 0.11871 |
| FoxA3 | chr7:15875216-15875625 | 11.2 | 15892300 | 16880 | 3'end | - | 0.5110125 |
| IGF1 | chr10:87838339-87838772 | 11.2 | 87833042 | 5513 | inside | + | 0.1272775 |
| krt1-1 | chr11:99860331-99860764 | 11.1 | 99871638 | 11091 | 3'end | - | 0.0991575 |
| Nfia | chr4:96799412-96799821 | 11.1 | 96752972 | 46644 | inside | + | 0.507335 |
| Gata6 | chr18:11097599-11097999 | 11.1 | 11097852 | 53 | 5'end | + | 0.637415 |
| tubb2 | chr13:33629476-33629860 | 11.1 | 33612495 | 17173 | 5'end | - | 0.099815 |
| TLE4 | chr19:13726074-13726483 | 11.1 | 13821845 | 95567 | inside | - | 0.972845 |
| Trf | chr9:103188434-103188843 | 11.1 | 103205727 | 17089 | inside | - | 0.032825 |
| CREB-H | chr10:81226628-81227012 | 11.1 | 81232760 | 5940 | inside | - | 0.277985 |
| ghrelin | chr6:114287173-114287557 | 11.0 | 114288467 | 1102 | inside | - | 0.2353425 |
