# Supplementary material for: Study of FoxA Pioneer Factor at Silent Genes Reveals Rfx-Repressed Enhancer at Cdx2 and a Potential Indicator of Esophageal Adenocarcinoma Development
Source: PLoS Genet. 2011 Sep 15;7(9):e1002277. doi: 10.1371/journal.pgen.1002277 (PMC3174211; doi:10.1371/journal.pgen.1002277)
Supplement: Table S3 — Primers for ChIP-qPCR. (DOCX) [file pgen.1002277.s008.docx]

**Supplemental Table 3: Primers for ChIP-qPCR**

| 1782 | Acvr2a_+2kb_F | GCAAGCTAAACAATGGTGTCTG |
| --- | --- | --- |
| 1783 | Acvr2a_+2kb_R | AGACCTGAGCAGGAGTTTACCA |
| 1950 | AFM_+42_F | GAAAGTCACATGATGGTCCTGA |
| 1951 | AFM_+42_R | TGCAAAGTTCTAAAGAGGCAAA |
| 1952 | AFM_p_F | ACCTAGAAATTTGCACCAGGAC |
| 1953 | AFM_p_R | TCATGCATTTAAAGGAGCAATG |
| 2517 | Afp_-5kb_F | TTAGCATCTTTTTGATGGCAGA |
| 2518 | Afp_-5kb_R | TCAAAGCGCAGTCCTAAGTACA |
| 1415 | Alb_+5Kb_F | TGCAGTCCTAGTCAGGGTGA |
| 1416 | Alb_+5Kb_R | CAGCTGGCTTGATGGCTAAA |
| 1519 | Alb_eG_F | GGGACGAGATGGTACTTTGTG |
| 1520 | Alb_eG_R | GATCAGTCCAAACTTCTTTCTG |
| 1784 | Arg1_+4kb_F | CAACACTGGTAGATGGTCAGGA |
| 1785 | Arg1_+4kb_R | TTCTCCTCAAGAGCCCTAAGTG |
| 1786 | Arg1_-3kb_F | GTTCTCCTTGCTGTATGGGTTC |
| 1787 | Arg1_-3kb_R | ATCTATCAGCCCCAAAAGGAAT |
| 1788 | Arg1_p_F | ATTCAAAGCCAAACTCACCTGT |
| 1789 | Arg1_p_R | AAAGATGTGCCCTCTGTCTTTT |
| 1790 | Bmp1_+4kb_F | GCAAGAGAGTTCAGGATGCTTT |
| 1791 | Bmp1_+4kb_R | GTGCTTCCTGCCTGGTTTATAG |
| 1794 | Cdh16_+13kb_F | AACTCGGTTTAGCTTCTGAACG |
| 1795 | Cdh16_+13kb_R | GAGGAACTCCTCCATGTGAGAC |
| 1792 | Cdh16_+16kb_F | CTCCTTCAGAAGTGGAGGAGTG |
| 1793 | Cdh16_+16kb_R | AAGCTCCAATCCCTGCTTTTAG |
| 1391 | CDX2_+3Kb_F | CCTGGGTTAGGGAGGTTTGT |
| 1392 | CDX2_+3Kb_R | TCTGTGTACACCACCCGGTA |
| 1393 | CDX2_+7Kb_F | CTGTTTGGACAGTGGAGCAA |
| 1394 | CDX2_+7Kb_R | ACAATGCCGACTTTTGAACC |
| 2291 | Cdx2_p_F | CTCCCCAACTTTTAAAATGCAA |
| 2292 | Cdx2_p_R | GGCCTTACGTGATTAACGAGTG |
| 1796 | Fapb2_-23kb_F | GTTTTGATGCTGTCTTGGTTTG |
| 1797 | Fapb2_-23kb_R | TCTGGGGCTTAGACTTCTTTTG |
| 1798 | Fapb2_-26kb_F | CACTCATCTCATTGGGATTCAA |
| 1799 | Fapb2_-26kb_R | AGGCTATAGAGGGAGCTTTGCT |
| 1800 | Fga_-23kb_F | GGGTTTCCTGGACCTTTGTAAC |
| 1801 | Fga_-23kb_R | ACTAACCGCCACAGCTACAAGT |
| 1746 | FoxA2_+5kb_F | ATCTCCCAGTAACTCAGGTGGA |
| 1747 | FoxA2_+5kb_R | TCTTAGCCCAGCTAAAGTCCAG |
| 1730 | FoxA2_+7kb_F | GAGGCATCCCTGCATTTATTT |
| 1731 | FoxA2_+7kb_R | TGAAATCTTTGCTTTGTGTGCT |
| 1728 | FoxA2_p_F | CCTGGTCGTCAGTTACCTCAGT |
| 1729 | FoxA2_p_R | GCACCTTGGATTTAACTGAAAA |
| 1726 | Gata4_+13kb_F | GAAAACATTGCAAAGTGGTGAA |
| 1727 | Gata4_+13kb_R | CTGATCGATAGCAACTGGTGAG |
| 1748 | Gata4_+29kb_F | ACGTGCTGACCTACCTCATTTT |
| 1749 | Gata4_+29kb_R | TCAACAGGTCCATACTGTGAGG |
| 1724 | Gata4_+40kb_F | TCCCATCACACTGTAATTGTCC |
| 1725 | Gata4_+40kb_R | CTAGGCAGGGATTAAGCAGCTA |
| 1802 | Gata6_+27_F | TGGGAGCTACTCTAAGCAGGAC |
| 1803 | Gata6_+27_R | GTACATCCCTGAAACCATCTCC |
| 1694 | Gata6_-1.7kp_F | GTCCGAAGCTTTAATGAATTGC |
| 1695 | Gata6_-1.7kp_R | GACTAGCAGCTGGAACAGGATT |
| 1696 | Gata6_-4.5kb_F | AGGGGCAAATTTAAATGGAACT |
| 1697 | Gata6_-4.5kb_R | CTCTCGAATCCCGATAAAGAAA |
| 1692 | Gata6_p_F | GCAGCTTGTAGAGAGCAGTTCC |
| 1693 | Gata6_p_R | ACAAAGGAGGAAACAACCGAAC |
| 1750 | GC_-11kb_F | GTTGTTTTCAGGGATGGGTAGA |
| 1751 | GC_-11kb_R | AAAGTATTGCTGCAGGTGGATT |
| 1732 | GC_-9kb_F | AAGGCTCAAAGTCCTAGTGCAA |
| 1733 | GC_-9kb_R | TGTGTGTGGGTCTTAAGTCTGG |
| 1752 | Hex_+11kb_F | AGTTTTTGGCTAAGGGGAGAAG |
| 1753 | Hex_+11kb_R | TATCTTGGTCCCTCAATCCACT |
| 1743 | Hex_+8kb_F | CCCTGGCAATCTGTAACTTTGT |
| 1742 | Hex_+8kb_F | CCTTCAAGGTTGCTTTGAATCT |
| 2525 | Hhex_p_F | TTCTACATCGACGACATCTTGG |
| 2526 | Hhex_p_R | AGGCTGGTGAAGGAGGAGTT |
| 1686 | Hnf4a_-5kb_F | TGACTAGAGGTCTGTCGAGTGC |
| 1687 | Hnf4a_-5kb_R | CCATGAAGCGTGTAACAGAAGA |
| 1684 | Hnf4a_-6kb_F | CTTTCCCAGCTTGAAAATGAAC |
| 1685 | Hnf4a_-6kb_R | CTCCAGGGCCTGAGTATATTTG |
| 1688 | Hnf4a_p_F | GACTATTAATGAGCGGGAGGTG |
| 1689 | Hnf4a_p_R | AACTGTCCTCTGGGAGACTCAG |
| 1804 | Hpxn_p_F | GCAGATGACATCACAGCAAATAA |
| 1805 | Hpxn_p_R | CACCACCTCTTGTCAGTATTCG |
| 1954 | Igf1_-1.2kb_F | GTGCTGTCATCACATGATTCCT |
| 1955 | Igf1_-1.2kb_R | GTGGTGATTGTCCTCAAATGAA |
| 1956 | Igf1_p_F | TAACTTTGCCAGAAGAGGGAGA |
| 1957 | Igf1_p_R | GCAAGCAGAAGAGGGATTTAGA |
| 1958 | Igfbp1_-3kb_F | GGAATCAAAGAGGAGTTTGGTG |
| 1959 | Igfbp1_-3kb_R | GTCCTTCCTCCTTTCCAAAGAT |
| 1960 | Igfbp1_p_F | TAGGTCTTTGATTTCCCCTGAA |
| 1961 | Igfbp1_p_R | GGGTAAAGGGATCAGGTTTTCT |
| 1808 | Krt20_-11kb_F | TTCACCCCATCTTTTTGTCTCT |
| 1809 | Krt20_-11kb_R | ATGTTTGCAATGTTGCTACAGG |
| 1806 | Krt4_+2kb_F | ACACAGCTCTTGCTTCACTCAG |
| 1807 | Krt4_+2kb_R | GGTTGCTACCACTAGGTTCTGG |
| 1754 | Lhx1_+10kb_F | CTCTCCTGTCTGCTCCTGACTT |
| 1755 | Lhx1_+10kb_R | AGGAAGAAGATTCCTGGGGTAG |
| 1738 | Lhx1_+13kb_F | TCAAGTGTTATCGGCTACATGC |
| 1739 | Lhx1_+13kb_R | GTCAGGGAGGTGTGAGAAAGAC |
| 1736 | Nanog_-14kb_F | GCCTACATGTACCTGTGGTCAA |
| 1737 | Nanog_-14kb_R | GAGTTTTGTGGGGCTTATTCAG |
| 1756 | Nanog_-15kb_F | GTGGAGGTCAGAGGACAAACTT |
| 1757 | Nanog_-15kb_R | GAGATCTGACACCCTCTTCTGG |
| 1810 | Nfia_+46kb_F | GGAGCAGTTGGAAGAAGAAAAG |
| 1811 | Nfia_+46kb_R | TGCCTGCCAAGAGTATGAACT |
| 1177 | Ngn3_-2kb-F | TCGCCTTAGGAGCAGGTGAT |
| 1178 | Ngn3_-2kb-R | TTGTAAAGCGGGATGCTTTG |
| 1962 | OC2_-14kb_F | CTACCTGTGCTGATACCGACAA |
| 1963 | Oc2_-14kb_R | GGACAGACTGCCTTTGATTTTC |
| 1964 | OC2_p_F | ACTGAATGAAGGCTGGCTACAC |
| 1965 | OC2_p_R | ATTGTCAGCTGCGGGATCAT |
| 1758 | Pax6_-14kb_F | AAAATAATTCCGTGGGAATGTG |
| 1759 | Pax6_-14kb_R | AAACTTAAGCCCTTTCCTTTGG |
| 1740 | Pax6_-17kb_F | AAAGAATGAAGGGGAGAAGGAC |
| 1741 | Pax6_-17kb_R | GTTCTCTTTTTGTCTGCCTGCT |
| 1682 | PDX_+11kb_F | TTTATGGCCCTTGTGAAGTAGG |
| 1683 | PDX_+11kb_R | GGGGACCTCATGTATCTGAAAA |
| 1547 | PDX_-2kb_F | GGAAATCCTTCCCTCAAGTTTT |
| 1548 | PDX_-2kb_R | GTAAATTGGCTTCCATCTCGAC |
| 1549 | PDX_-6kb_F | CTCTTCCTGATTCCCTGAAGTC |
| 1550 | PDX_-6kb_R | ACTAAGAGTGCTCTGGGCTCTG |
| 1397 | PDX_p_F | GGATCAGGCGACTGAGAGAG |
| 1398 | PDX_p_R | TTTACCCTGGAGCCATCATT |
| 1968 | Ppara_-2.8_F | ACACAGGATAGGAGGCTTTGAA |
| 1969 | Ppara_-2.8_R | GGCTCGTCTATGTTTACCTTGC |
| 1966 | Ppara_36kb_F | TTTGTGGGCATTTTCTACACTG |
| 1967 | Ppara_36kb_R | TGAGTTCAGCTAGGCAAAGGAT |
| 1970 | Ppara_p_F | ACAGTGAGGTGGGTGGACAG |
| 1971 | Prara_p_R | CTTCCTAGCGTGTGCCCTCT |
| 1744 | Ptch1_-11kb_F | TTGAGGACCAAAGGGGTAGATA |
| 1745 | Ptch1_-11kb_R | GGTTGGTCATTTCCTCTGACTC |
| 1760 | Ptch1_-14kb_F | CCGTAAAAACACAAACAGGACA |
| 1761 | Ptch1_-14kb_F | TGGAACCTGCTTTCTAGCTCTC |
| 1680 | Ptf1a_-1.7kb_F | TTAAACCCCAGAGTCAAGAAGC |
| 1681 | Ptf1a_-1.7kb_R | CCCTGTCTAAAACAAGGGTGAG |
| 1677 | Ptf1a_-12kb_F | GCTTGCCATTAGGGAAACATAG |
| 1676 | Ptf1a_-12kb_R | AGAGTCCTCATTCTCTGCCTTG |
| 1812 | Serpina1d_+4kb_F | AGATCATAAAGGGCAATCTTGG |
| 1813 | Serpina1d_+4kb_R | TCAAGTCTTGCTTCACTTCAGC |
| 1734 | Sftpb_-10kb_F | AGGAACACTGGACTGACAAACA |
| 1735 | Sftpb_-10kb_R | CTGCTGAGGAAATGCTGCTAC |
| 1762 | Sftpb_-7kb_F | AGCTTTTGGGGTTTGTATGAAA |
| 1763 | Sftpb_-7kb_R | GTGTCAAACTGGACCCTTGAGT |
| 2521 | Sftpb_p_F | GGACATCATCAGTGTCACCAGT |
| 2522 | Sftpb_p_R | CATACCAGGTTTACGTCCCATT |
| 1814 | Tcf2_+26kb_F | GGAAGACCTTTCTCCTTCTCGT |
| 1815 | Tcf2_+26kb_R | AACTAGTCAGGGCTCTGTCACC |
| 1976 | Tle1_+2kb_F | CCCCTAATGGACTTTGTCTCAG |
| 1977 | Tle1_+2kb_R | GCGCTTCGTTTATTTATTTTGC |
| 1978 | Tle1_p_F | CACCGCAAAGTTCTCACGAC |
| 1979 | Tle1_p_R | TGAAACCGCGTGTTAATGTAAG |
| 1291 | TTR_+8kb_F | CAGGATCTTGCCAAAGCAGT |
| 1292 | TTR_+8kb_R | AGAATGCTTCACGGCATCTT |
| 1206 | TTR_p_F | AGCGAGTGTTCCGATACTC |
| 1205 | TTR_p_R | ACCCCCTCCTTCCAACCCA |
| 1820 | Vegfa_-20kb_F | TCAAGAGGGGAAAGTTTGAAGA |
| 1821 | Vegfa_-20kb_R | GGCACACACACGTGTCAGACT |
| 1818 | Vegfa_-23kb_F | AAAGGAGAGGGGTCTAAGCAAC |
| 1819 | Vegfa_-23kb_R | TCGAGATGGAATGTCAGAGAGA |
|  |  |  |
|  |  |  |
| HUMAN CHIP PRIMERS | |  |
| 2128 | Cdx2_+7kb_human_R | ACAATGTCGACTTTTGAACC |
|  | cdx2_+7kb_F, TTF1 +2kb same as mouse primers | |
