# Supplementary material for: Study of FoxA Pioneer Factor at Silent Genes Reveals Rfx-Repressed Enhancer at Cdx2 and a Potential Indicator of Esophageal Adenocarcinoma Development
Source: PLoS Genet. 2011 Sep 15;7(9):e1002277. doi: 10.1371/journal.pgen.1002277 (PMC3174211; doi:10.1371/journal.pgen.1002277)
Supplement: Table S4 — Taqman Probe Ids. (DOCX) [file pgen.1002277.s009.docx]

**Supplementary Table 4: Taqman Probe Ids**

| Gast-Mm00772211_g1 | Target |
| --- | --- |
| Pax6-Mm00443072_m1 | Target |
| Cp-Mm00432654_m1 | Target |
| Sox17-Mm00488363_m1 | Target |
| Krt1-1-Mm00657991_gH | Target |
| Ptpro-Mm00478480_m1 | Target |
| Ppara-Mm00440939_m1 | Target |
| Nr1i3-Mm00437986_m1 | Target |
| Apoa1-Mm00437569_m1 | Target |
| Srebf1-Mm00550338_m1 | Target |
| 18S-Hs99999901_s1 | Endogenous Control |
| Cebpa-Mm00514283_s1 | Target |
| Cdh1-Mm00486906_m1 | Target |
| Apoe-Mm00437573_m1 | Target |
| Aldob-Mm00523296_m1 | Target |
| Proc-Mm00435966_m1 | Target |
| Nr2f2-Mm00772789_m1 | Target |
| Arg1-Mm00475988_m1 | Target |
| Cebpb-Mm00843434_s1 | Target |
| Slco1a4-Mm00453126_m1 | Target |
| Vegfa-Mm00437304_m1 | Target |
| Foxa2-Mm00839704_mH | Target |
| Hprt1-Mm00446968_m1 | Target |
| Pck1-Mm00440636_m1 | Target |
| Cebpb-Mm00843434_s1 | Target |
| Nfib-Mm00500784_m1 | Target |
| Foxa1-Mm00484713_m1 | Target |
| Cyp7a1-Mm00484152_m1 | Target |
| Sbsn-Mm00552057_m1 | Target |
| Prox1-Mm00435969_m1 | Target |
| Gata6-Mm00802636_m1 | Target |
| Nfix-Mm00477796_m1 | Target |
| Onecut1-Mm00839394_m1 | Target |
| Pah-Mm00500918_m1 | Target |
| Sftpc-Mm00488144_m1 | Target |
| Sftpb-Mm00455681_m1 | Target |
| Smad4-Mm00484724_m1 | Target |
| Tcirg1-Mm00469395_g1 | Target |
| 9030623C06Rik-Mm00508106_m1 | Target |
| Lhx1-Mm00521776_m1 | Target |
| Afp-Mm00431715_m1 | Target |
| Atp4b-Mm00437657_m1 | Target |
| Clcn1-Mm00658624_m1 | Target |
| Igf1-Mm00439559_m1 | Target |
| Dlk1-Mm00494477_m1 | Target |
| Gck-Mm00439129_m1 | Target |
| Krt1-19-Mm00492980_m1 | Target |
| Slco1c1-Mm00451845_m1 | Target |
| Cdx2-Mm00432449_m1 | Target |
| Foxa3-Mm00484714_m1 | Target |
| Gata4-Mm00484689_m1 | Target |
| Igfbp1-Mm00833447_m1 | Target |
| Onecut2-Mm00815708_s1 | Target |
| Tle3-Mm00437097_m1 | Target |
| Tcf2-Mm00447452_m1 | Target |
| Afm-Mm00446866_m1 | Target |
| Sst-Mm00436671_m1 | Target |
| Fabp1-Mm00444340_m1 | Target |
| Lcat-Mm00500505_m1 | Target |
| Tat-Mm00455392_m1 | Target |
| Kit-Mm00445212_m1 | Target |
| G6pc-Mm00839363_m1 | Target |
| Nfic-Mm00476357_m1 | Target |
| Slc10a1-Mm00441421_m1 | Target |
| Nfic-Mm00546636_s1 | Target |
| Mat1a-Mm00522563_m1 | Target |
| Gnal-Mm00622607_m1 | Target |
| Serpina1d-Mm00842094_mH | Target |
| Tubb2a-Mm00809562_s1 | Target |
| Slc2a2-Mm00446224_m1 | Target |
| Nfia-Mm00447981_m1 | Target |
| Fgl1-Mm00521175_m1 | Target |
| Hnf4a-Mm00433964_m1 | Target |
| Saa3-Mm00441203_m1 | Target |
| Hdc-Mm00456104_m1 | Target |
| Fgfr2-Mm00438941_m1 | Target |
| Ipf1-Mm00435565_m1 | Target |
| Fabp2-Mm00433188_m1 | Target |
| Notch2-Mm00803077_m1 | Target |
| Tle1-Mm00495643_m1 | Target |
| Gli3-Mm00492333_m1 | Target |
| Hes1-Mm00468601_m1 | Target |
| Cdh16-Mm00483196_m1 | Target |
| Ttr-Mm00443267_m1 | Target |
| Xbp1-Mm00457359_m1 | Target |
| Tff3-Mm00495590_m1 | Target |
| Alb1-Mm00802090_m1 | Target |
| Acvr2a-Mm00431657_m1 | Target |
| Muc1-Mm00449604_m1 | Target |
| Trf-Mm00446708_m1 | Target |
| Dpp4-Mm00494548_m1 | Target |
| Dbp-Mm00497539_m1 | Target |
| Hpxn-Mm00457510_m1 | Target |
| Fga-Mm00802584_m1 | Target |
| Hhex-Mm00433954_m1 | Target |
| Ptch1-Mm00436026_m1 | Target |
